# Epistasis reduces fitness costs of influenza A virus escape from stem-binding antibodies

**DOI:** 10.1073/pnas.2208718120

**Published:** 2023-04-17

**Authors:** Chung-Young Lee, Vedhika Raghunathan, C. Joaquin Caceres, Ginger Geiger, Brittany Seibert, Flavio Cargnin Faccin, L. Claire Gay, Lucas M. Ferreri, Drishti Kaul, Jens Wrammert, Gene S. Tan, Daniel R. Perez, Anice C. Lowen

**Affiliations:** ^a^Department of Microbiology and Immunology, Emory University School of Medicine, Atlanta, GA 30322; ^b^Department of Microbiology, School of Medicine, Kyungpook National University, Daegu 41944, The Republic of Korea; ^c^Department of Population Health, College of Veterinary Medicine, University of Georgia, Athens, GA 30602; ^d^J. Craig Venter Institute, La Jolla, CA 92037; ^e^Division of Infectious Diseases, Department of Pediatrics, Emory University School of Medicine, Atlanta, GA 30322; ^f^Division of Infectious Disease, Department of Medicine, University of California San Diego, La Jolla, CA 92093; ^g^Emory-University of Georgia Center of Excellence for Influenza Research and Surveillance, Atlanta, GA 30322

**Keywords:** influenza A virus, HA stem, evolution, antigenic escape, epistasis

## Abstract

The hemagglutinin (HA) stem region is a major target of universal influenza vaccine efforts owing to the presence of highly conserved epitopes across multiple influenza A virus (IAV) strains and subtypes. To explore the potential impact of vaccine-induced immunity targeting the HA stem, we examined the fitness effects of viral escape from stem-binding broadly neutralizing antibodies (stem-bnAbs). Recombinant viruses containing each individual antibody escape substitution showed diminished replication compared to wild-type virus, indicating that stem-bnAb escape incurred fitness costs. A second-site mutation in the HA head domain (N129D; H1 numbering) reduced the fitness effects observed in primary cell cultures and likely enabled the selection of escape mutations. Functionally, this putative permissive mutation increased HA avidity for its receptor. These results suggest a mechanism of epistasis in IAV, wherein modulating the efficiency of attachment eases evolutionary constraints imposed by the requirement for membrane fusion. Taken together, the data indicate that viral escape from stem-bnAbs is costly but highlights the potential for epistatic interactions to enable evolution within the functionally constrained HA stem domain.

## Significance

The stem domain of the influenza A virus hemagglutinin protein is highly conserved, making it an ideal target for vaccines designed to confer protection against diverse strains. This conservation is thought to be a consequence of the essential functions of the stem region in mediating viral entry into cells. We evaluated the potential for the stem domain to evolve to evade immune restriction while maintaining this functionality. We found that functional interactions between different regions of hemagglutinin can facilitate this evolution. Our results document the potential for influenza A virus evolution to mediate escape from HA stem targeting vaccines and therefore indicate a need to incorporate monitoring of viral antigenic evolution into evaluations of candidate universal influenza vaccines.

Hemagglutinin (HA) is the surface glycoprotein of influenza A virus (IAV) that mediates viral attachment and entry into host cells. The globular head of HA contains the receptor binding site (RBS) that binds to sialylated glycans on respiratory epithelial cells, allowing their uptake by endocytosis (1, 2). During the endocytic pathway, an acidic endosomal environment triggers the release of the fusion peptide from within the stem domain of the HA trimer, leading to fusion between viral and endosomal membranes and release of the viral ribonucleoproteins into the cytoplasm (3–5).

The globular head of HA is the main target of neutralizing antibodies elicited by vaccination or infection (6, 7). These antibodies typically act by blocking the interaction between HA and sialylated glycan receptors, inhibiting viral attachment (1, 5, 8). However, selective pressure leads to antigenic changes in circulating viruses, causing reduction in or loss of neutralization (9–11). This variability in HA lowers the cross-protection of humoral immune responses elicited by traditional vaccines (12). The resultant narrow specificity of influenza vaccines necessitates their annual updating and gives the potential for mismatch between circulating strains and those included in the vaccine. Therefore, substantial efforts have been invested to develop vaccines targeting more conserved regions of IAV, with the goal of conferring broad protection (7).

The stem region in HA has considerably higher sequence conservation than the globular head. The stem consists of the N- and C-terminal regions of HA1 and most of HA2. Generally, antibodies to the stem are harder to elicit but have greater breadth than head-binding antibodies, neutralizing a broader range of IAVs (6, 13–17). In addition, the stem region is thought to be inherently less permissive to viral escape because it is essential to stabilize the HA trimeric structure and to drive viral fusion through HA conformational change (18, 19). Therefore, the stem has been regarded as a promising target for universal vaccines and antiviral therapeutics (7). Several stem-targeted, broadly reactive, neutralizing antibodies (stem-bnAbs) have been isolated from infected or immunized individuals since the first stem-bnAb was isolated in 1993 (14). These antibodies are categorized based on the breadth of their neutralization potential to group 1 IAVs (14, 15, 20–22), group 2 IAVs (17, 23), pan-IAV (16, 24), or IAVs and influenza B viruses (25).

High conservation of the HA stem domain within circulating viruses suggests that its evolution is limited due to functional or structural constraints. Laboratory-based studies in which selection on the stem domain is imposed experimentally can give valuable insight. In this context, escape mutations against stem-bnAbs have been identified in different subtypes of IAV (15, 26–32). These mutations typically abolish or reduce antibody binding (28–32), although in one study escape was instead mediated by enhanced HA-mediated fusion activity (31). Mutation in neuraminidase (NA) was also found to contribute to escape from stem-bnAbs, likely by modulating the balance between HA and NA functions in viral attachment and release (26). Irrespective of the mechanism of escape, the mutations characterized typically incur a fitness cost, attenuating viral replication in either in vitro or in vivo models (26, 28–31). This attenuation is likely associated with lower mutational tolerance of the HA stem domain compared to the HA head domain (33), even under immunological pressure (34).

In this study, we monitored the evolution of influenza A/Netherlands/602/2009 (NL09) virus, a 2009 pandemic H1N1 strain, in the presence of two different stem-bnAbs (70-1F02 and 05-2G02). We identified several individual escape mutations in this process. As expected, all isolated escape mutations carried viral fitness costs. However, these costs were diminished by a second-site mutation, N129D (N133D in H3 numbering), which was found during virus passage prior to fixation of the escape mutations. N129D increased virus binding to receptors, resulting in enhanced replication of the escape mutant viruses in primary human cell cultures. These data emphasize the importance of positive epistasis for viral evolution and suggest that epistasis may enable antigenic drift under pressure imposed by HA stem-targeted vaccines.

## Results

### Identification of Mutations Allowing Escape from Two Stem-bnAbs.

To isolate escape variants against stem-bnAbs, A/Netherlands/602/2009 (H1N1) (NL09) virus was serially passaged ten times with either 70-1F02 or 05-2G02 stem-bnAbs as described before (35). 70-1F02, derived from an individual naturally infected with a pandemic H1N1 IAV, has demonstrated cross-reactivity to H1 and H5 subtype strains (20, 36). 05-2G02, isolated from a healthy subject immunized with the inactivated 2009 pandemic H1N1 vaccine, has broad reactivity with strains of group 1 and group 2 HAs (24, 36). As a control, we also passaged NL09 virus without stem-bnAbs ten times. Each passage regimen was carried out in triplicate.

**Fig. 1.**
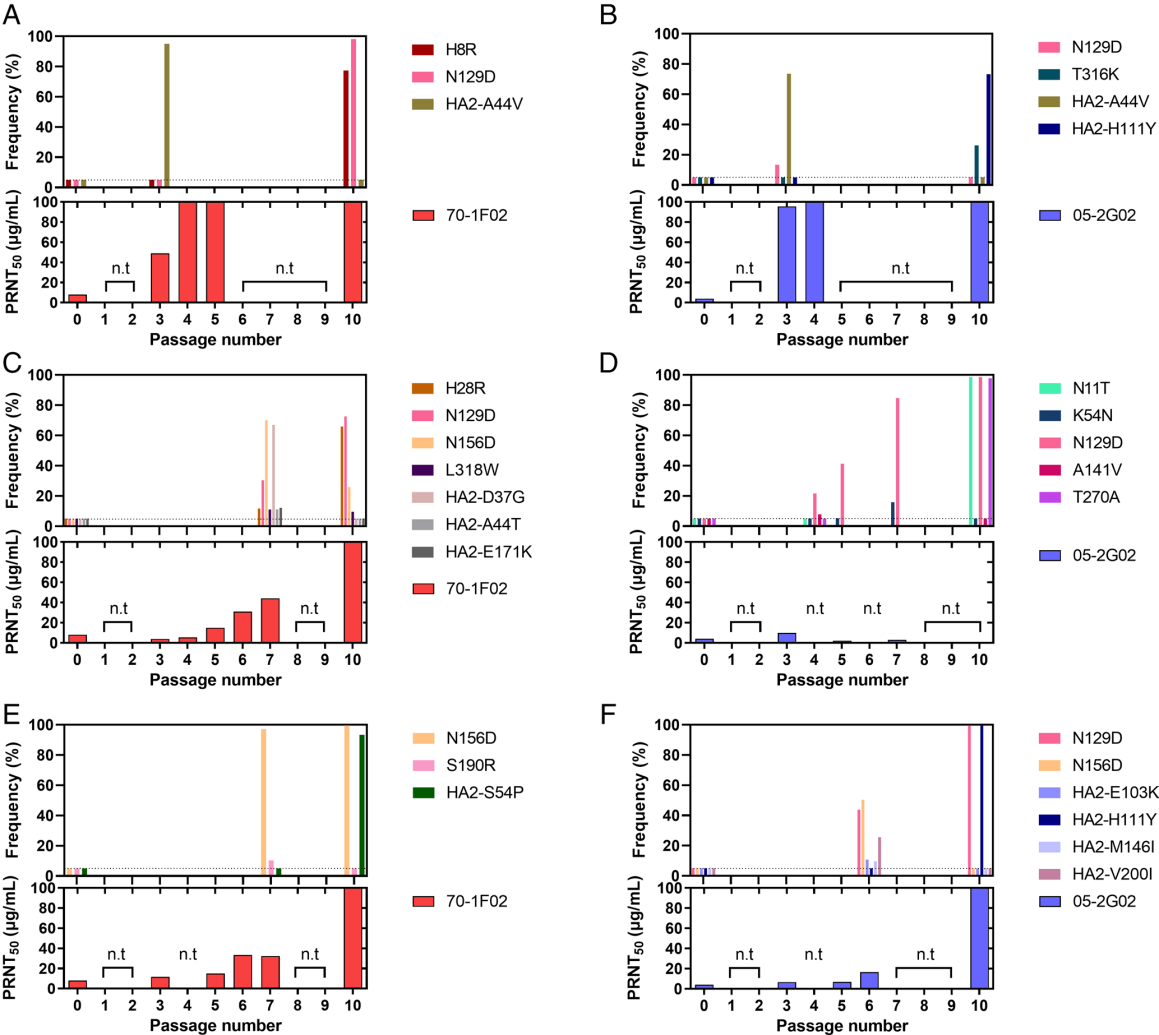
Dynamics of mutation frequency and resistance of passaged NL09 virus populations to stem-bnAbs. NL09 virus was combined with (*A*, *C*, and *E*) 70-1F02 or (*B*, *D*, and *F*) 05-2G02 antibody and serially passaged 10 times with increasing concentrations of antibody. A single replicate was performed with an initial concentration of 20 μg/mL (*A* and *B*), 2 μg/mL (*C* and *D*), or 1 μg/mL (*E* and *F*) of 70-1F02 or 05-2G02, for a total of three replicates per stem-bnAb. n.t. = not tested; the dotted line indicates the limit of detection (5%). The earliest passaged populations that showed PRNT_50_ titers >40 µg/mL or P7 populations were sequenced. In panel *F*, the P6 variant was sequenced instead of the P7 variant because the titer of the P7 variant was not sufficient for sequencing. Amino acid positions are indicated with H1 numbering.

To assess the sensitivity of the passaged virus populations to stem-bnAbs, 50% plaque reduction neutralizing titers (PRNT_50_) were measured (Fig. 1 and *SI Appendix*, Fig. S1). The PRNT_50_ titers of viral populations passaged with stem-bnAbs increased with passage number and five of six passage-ten populations showed titers of more than 100 µg/mL (Fig. 1).

**Fig. 2.**
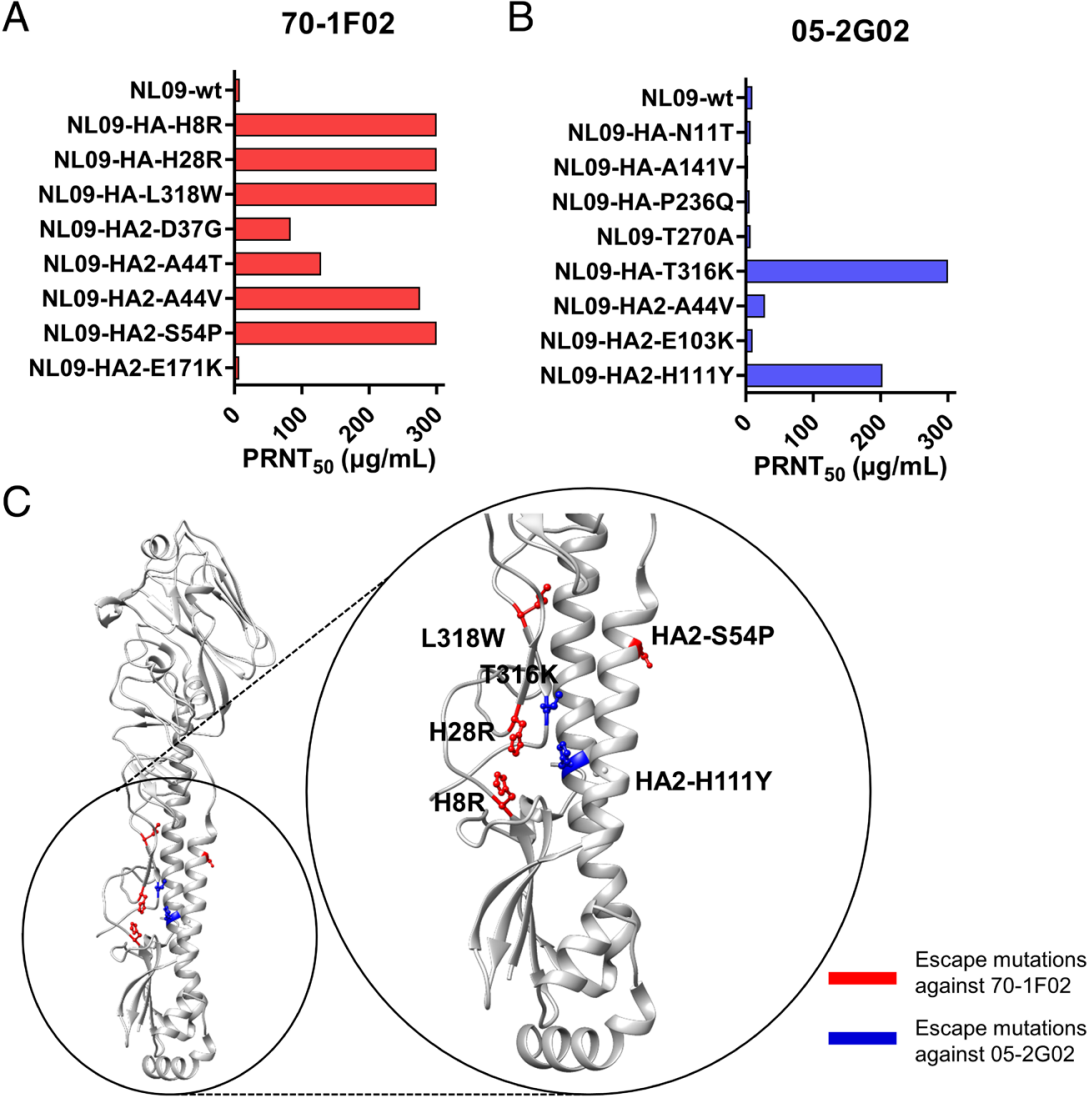
Identification of stem-bnAb escape mutations. (*A* and *B*) PRNT_50_ titers of recombinant NL09 viruses harboring individual mutations found in the passaged NL09 variants. Concentrations of (*A*) 70-1F02 and (*B*) 05-2G02 antibodies leading to a 50% reduction in plaque counts are shown. (*C*) Locations of selected escape mutations on the HA of A/California/04/2009 (H1N1) (PDB ID 3LZG) (39).

To identify and monitor the dynamics of escape mutations, deep sequencing was conducted on virus populations derived from both early and late passages. In addition, to confirm the frequency of mutations and assess their cooccurrence, we sequenced six plaque-purified clones per sample (*SI Appendix*, Fig. S2 and Datasets S1–S3). The results revealed that, within each passaged lineage, a single variant harboring one to three mutations became dominant. Each variant contained distinct mutations although some were shared (Fig. 1). The substitution N129D was dominant in four out of six antibody-selected populations and one out of four controls. H8R (H18R in H3 numbering) and H28R (H38R in H3 numbering) in HA1 and S54P in HA2 became dominant in populations passaged with 70-1F02. By contrast, N11T (N21T in H3 numbering) and T270A (T272A in H3 numbering) in HA1 and H111Y in HA2 became dominant in populations passaged with 05-2G02. To identify single mutations that confer antibody escape, we generated recombinant NL09 viruses bearing the individual mutations detected in the passaged virus variants and measured viral resistance to antibody (Fig. 2 *A* and *B*). The substitutions, such as N129D, that were found in both antibody-selected and control populations were excluded from this analysis. The recombinant viruses harboring H8R, H28R, L318W (L320W in H3 numbering), or HA2-S54P are highly resistant to 70-1F02 antibody, showing PRNT_50_ titers of more than 300 µg/mL. Similarly, the mutant viruses harboring T316K (T318K in H3 numbering) or HA2-H111Y are highly resistant to 05-2G02 antibody, showing titers of >300 or 202.9 µg/mL, respectively. In general, mutations conferring high PRNT_50_ titers coincide with those that dominated in passage ten populations, suggesting that their phenotypic effects led to their positive selection. There was one exception to this generalization, however: N11T and T270A, which were near fixation at passage 10, did not confer resistance to 05-2G02 antibody neutralization when introduced individually or in combination (Fig. 2 and *SI Appendix*, Fig. S3). While the basis for their positive selection is not clear, it is notable that N11T is expected to remove a glycosylation site that is known to modulate the efficiency of cleavage activation in the context of an H5 subtype HA (37, 38).

### The Escape Mutations Decrease Antibody Binding to NL09 HA.

**Fig. 3.**
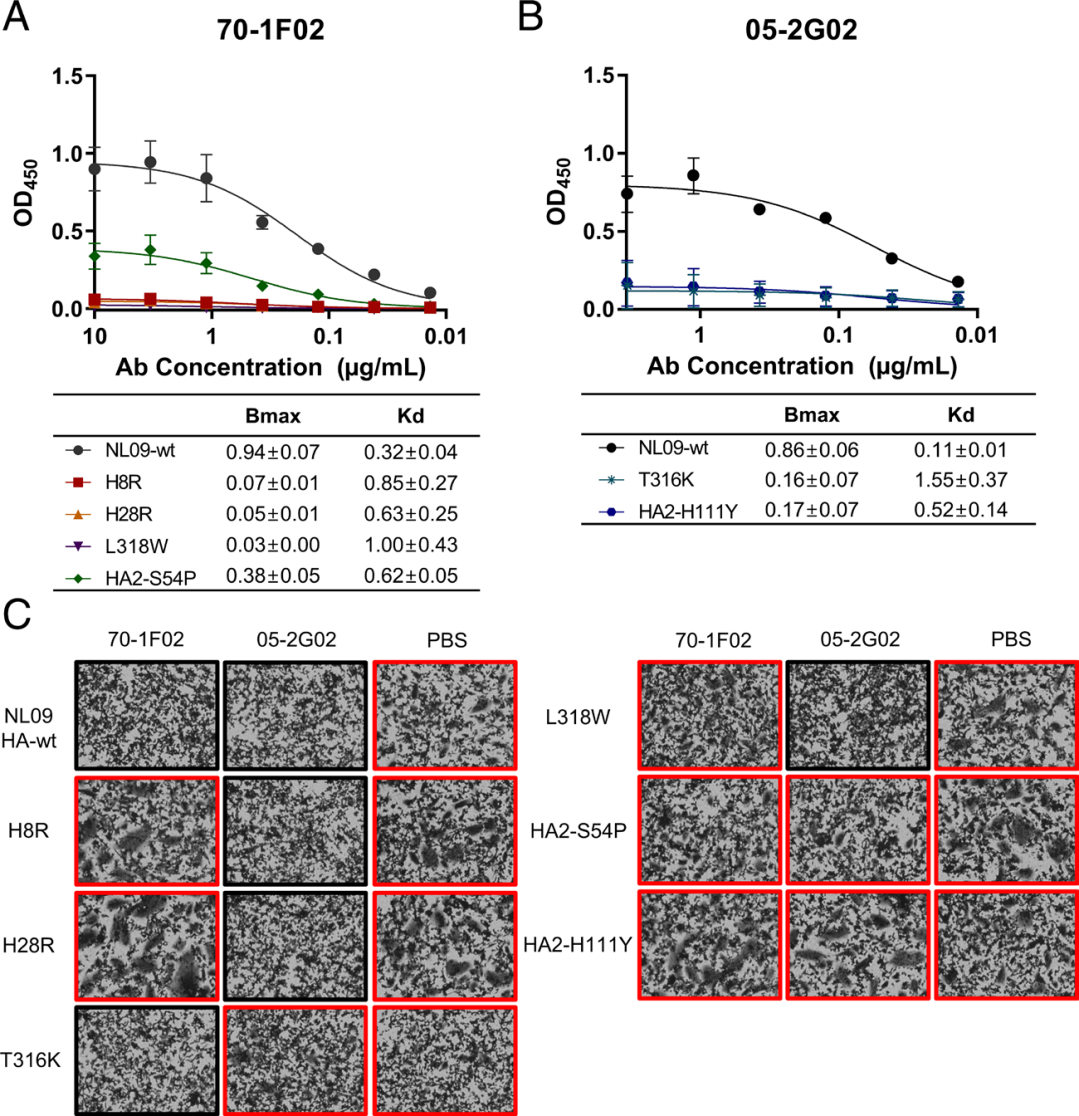
By diminishing antibody binding, the identified escape mutations enable HA-membrane fusion in the presence of stem-bnAbs. (*A* and *B*) Binding between mutant HA and the stem-bnAbs (*A*) 70-1F02 or (*B*) 05-2G02. The mean and SD of four independent experiments are plotted. Bmax and Kd values with 95% CI were calculated using GraphPad Prism 9.5.0. (*C*) HA-mediated membrane fusion was evaluated by monitoring for syncytia formation at low pH. Red rectangles indicate that syncytia formation was observed.

To investigate the mechanisms by which the escape mutations in HA confer resistance, we first mapped them onto a pandemic H1N1 HA structure (PDB ID 3LZG). The mutations conferring high resistance to the stem-bnAbs are located in the HA stem region (Fig. 2*C*). Among these mutations, L318W and HA2-S54P are buried within the monomer interior and H8R and H28R are present within the 70-1F02 epitope (36). Because a common strategy of escape is to abolish or reduce binding between antigen and antibody, we next evaluated the impact of the escape mutations on antibody binding. An enzyme-linked immunosorbent assay (ELISA)-based assay was used in which NL09 HA proteins harboring an individual escape mutation were overexpressed in adherent cells. HA protein expression was confirmed quantitatively using an HA head-specific antibody (1009-3E04) (*SI Appendix*, Fig. S4). Both 70-1F02 and 05-2G02 stem-bnAbs bound to the cells expressing NL09 HA-wt (Fig. 3 *A* and *B*). However, binding to 70-1F02 was essentially abolished by H8R, H28R, and L318W mutations and significantly reduced by the HA2-S54P mutation. Similarly, binding to 05-2G02 was disrupted by T316K and HA2-H111Y mutations.

Next, we investigated whether the loss of interaction between HA and antibody affects viral fusion activity using a syncytia formation assay. In NL09 HA-wt, both stem-bnAbs inhibited fusion (Fig. 3*C*). Conversely, cells overexpressing mutant HA proteins demonstrated syncytia formation in the presence of stem-bnAb. Notably, the HA2-S54P and H111Y mutants formed syncytia in the presence of both 07-1F02 and 05-2G02 and showed increased resistance to both antibodies (*SI Appendix*, Fig. S5), although these mutants were selected by either 70-1F02 or 05-2G02. These data indicate that each escape mutation examined acts by interrupting the binding of stem-bnAbs, enabling functional HA-membrane fusion in the presence of antibody.

### The Escape Mutations Confer Fitness Defects.

**Fig. 4.**
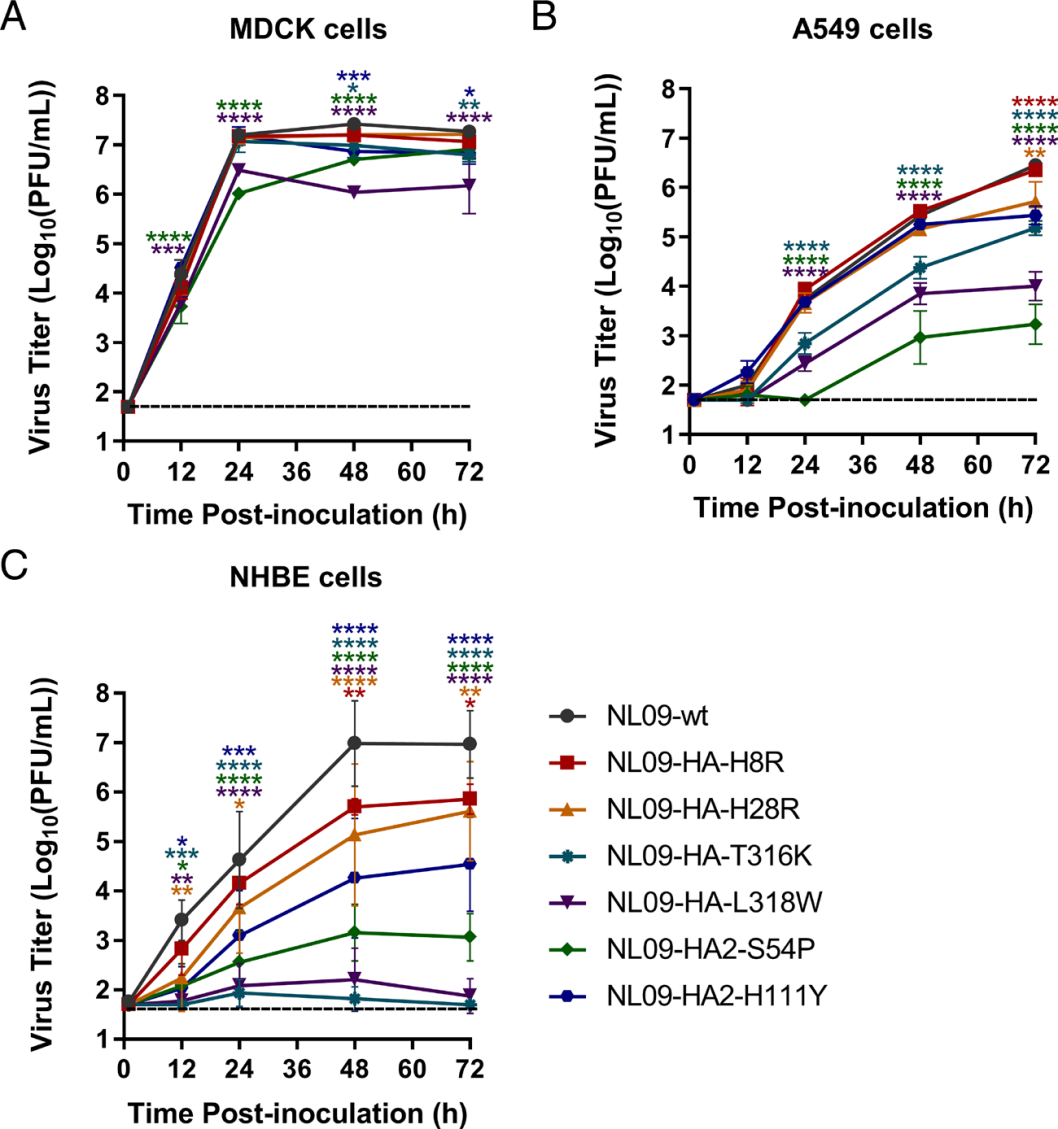
Replication of recombinant viruses harboring individual escape mutations. (*A*) MDCK cells were infected at an MOI of 0.002 PFU/cell; (*B*) A549 cells and (*C*) NHBE cells were infected with MOI = 0.01 PFU/cell. The mean and SD of at least three independent experiments are plotted. Statistical significance was assessed by two-way ANOVA with Sidak’s multiple comparisons test. ****, < 0.0001; ***, < 0.001; **, < 0.01; *, < 0.05.

Given the high conservation of the HA stem domain, mutations in the stem were expected to carry fitness costs. To test for fitness effects, we measured the growth kinetics of NL09 viruses harboring the individual escape mutations in Madin–Darbycanine kidney (MDCK), A549, and Normal human bronchial epithelial (NHBE) cells. In MDCK cells, the NL09 recombinant viruses carrying the individual escape mutations replicated comparably to NL09-wt virus, although viruses harboring T316K, L318W, HA2-S54P, or HA2-H111Y were slightly attenuated (Fig. 4*A*). Notably, stronger phenotypes were observed in A549 and NHBE cells (Fig. 4 *B* and *C*). In A549 cells, the viruses harboring T316K, L318W, and HA2-S54P replicated poorly compared to NL09-wt virus, while replication of NL09-HA-H8R, NL09-HA-H28R, and NL09-HA2-H111Y viruses was comparable to NL09-wt. In NHBE cells, all six mutant viruses were significantly attenuated, although H8R, H28R, and HA2-H111Y were more fit than the other mutant viruses. Collectively, the impact of the single escape mutations on viral fitness varied widely and was most readily detected in NHBE cells.

**Fig. 5.**
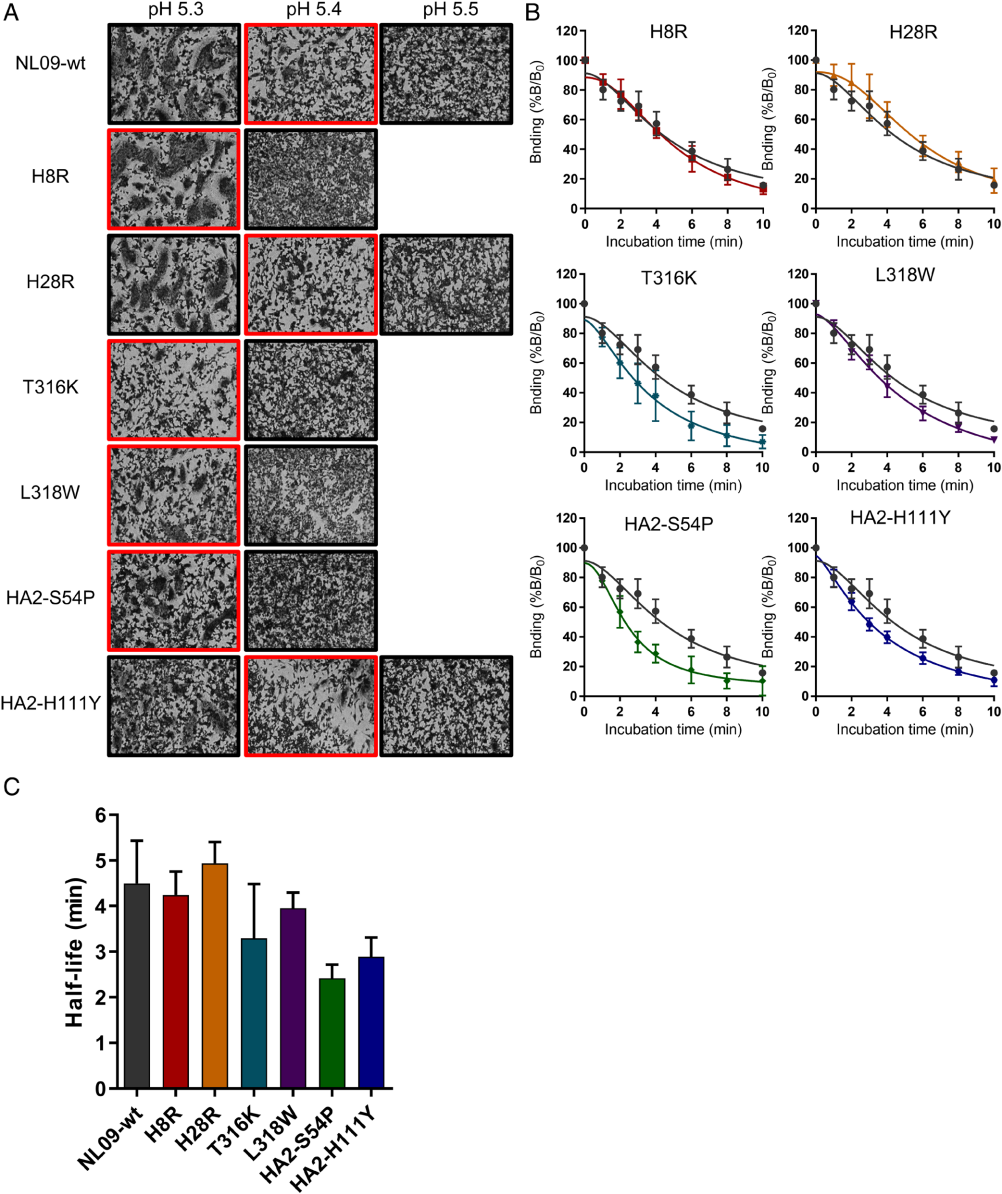
The effect of escape mutations on virus and membrane fusion. (*A*) Fusion pH of NL09 HA harboring individual escape mutations was measured by syncytium formation. Red rectangles mark the highest pH at which syncytia were observed. (*B*) Kinetics of low pH-induced conformational change of NL09 HA proteins containing single escape mutations, as measured by ELISA with a conformation-sensitive antibody. The mean and SD of five independent replicates are plotted. (*C*) Time to 50% dissociation of the conformation-sensitive antibody (half-life). The mean and SD of five independent replicates are plotted.

These findings prompted us to examine mechanism by which the escape mutations impact viral fitness. Owing to their location in the HA stem and the stem’s role in HA-mediated membrane fusion, we assessed pH of fusion and fusion kinetics. First, a syncytia formation assay was employed to measure the pH at which fusion takes place (Fig. 5). The HAs harboring the individual escape mutations led to membrane fusion at pH 5.3 or 5.4, a minor difference from NL09-wt HA. Next, fusion kinetics of the mutant HAs were evaluated. For this purpose, a head-specific antibody that is completely detached by acid-induced HA conformational change (*SI Appendix*, Fig. S6) was used to monitor HA conformation for 10 min in 2-min intervals. The half-life for antibody detachment from NL09-wt HA was 4.5 min (Fig. 5 *B* and *C*). T316K, L318W, HA2-S54P, and HA2-H111Y mutations decreased the half-life (to 3.3, 4.0, 2.4, and 2.9 min, respectively), indicating that these mutations destabilize the prefusion HA. In contrast, HAs carrying H8R (half-life = 4.2 min) or H28R (half-life = 4.9 min) exhibited similar fusion kinetics to NL09-wt HA. With the exception of HA2-H111Y, the mutations that hastened the fusion event were also seen to have the strongest fitness effects, suggesting that HA destabilization accounts for the attenuation of replication observed. By contrast, the strong effect of HA2-H111Y on fusion kinetics was associated with a more modest reduction of viral replication, potentially due to uncharacterized functional effects of this amino acid change.

Since the escape mutations were selected under antibody treatment, we evaluated their fitness relative to NL09-wt in the presence of stem bnAbs. NHBE cells were pretreated with 1 PRNT_50_ of the stem-bnAbs or phosphate-buffered saline (PBS), then inoculated with multiplicity of infection (MOI) = 1 PFU/cell of NL09-wt or the single escape mutant viruses. As expected, the replication of NL09-wt virus significantly decreased in the presence of 07-1F02 or 05-2G02 antibodies (*SI Appendix*, Fig. S7). In contrast, the escape mutant viruses harboring H8R, H28R, or HA2-H111Y replicated similarly with or without the antibodies, confirming their resistance to the relevant immunological pressure.

### N129D, a Potential Permissive Mutation, Enhances HA Binding to Receptors.

In addition to the escape mutations, four out of six passage 10 populations have a common second site mutation (N129D, H1 numbering) and one out of six has another second site mutation (N156D, H1 numbering, N159D in H3 numbering) in the HA globular head domain. Residue 129 is located in 130-loop, between Ca2 and Sa antigenic sites, and residue 156 lies within the Sa antigenic site (*SI Appendix*, Fig. S8*A*). They are close to the RBS and both have been reported to affect antigenicity and receptor binding affinity (40–43). Of note, since 2019, virus strains harboring an N129D mutation have increased in frequency and become dominant within the circulating 2009 pandemic H1N1 lineage (*SI Appendix*, Fig. S8*B*). Given these prior observations, we hypothesized that these mutations may act as permissive mutations, enabling the selection of escape mutations in the stem domain. Due to the proximity of N129D and N156D within the HA structure, their similar functional effects, and the higher prevalence of N129D compared to N156D among the escape variants, we chose to focus on the N129D mutation for further analysis.

First, we evaluated the impact of HA N129D on sensitivity to stem-bnAbs and HA fusion potential. The recombinant virus carrying N129D showed similar PRNT_50_ titer to NL09-wt virus (*SI Appendix*, Fig. S8), suggesting that N129D does not contribute directly to bnAb escape. Similarly, fusion pH and fusion kinetics of NL09-HA-N129D virus were indistinguishable from those exhibited by NL09-wt virus (*SI Appendix*, Fig. S8). Thus, N129D is unlikely to mitigate the effects on fusion brought about by mutations in the HA stem.

**Fig. 6.**
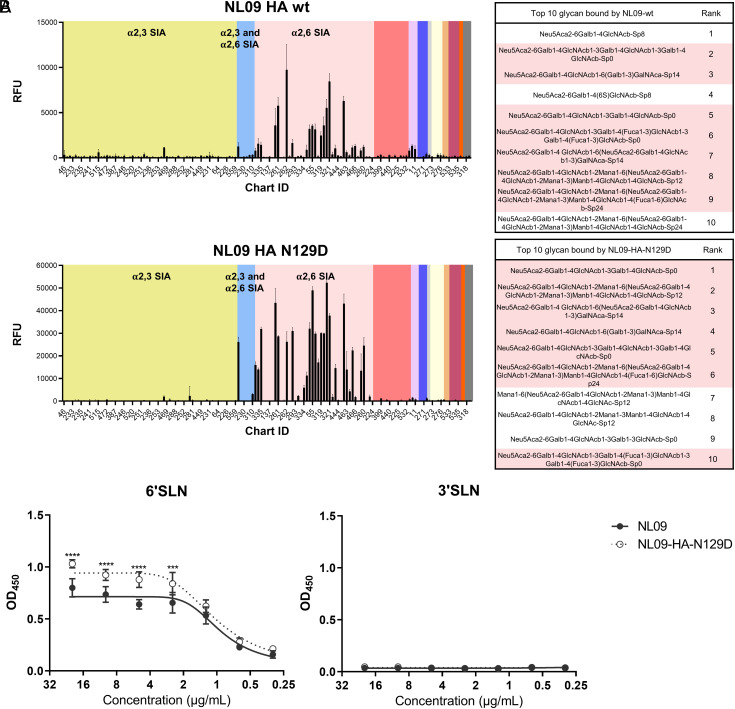
HA N129D does not markedly alter receptor specificity but enhances binding to human-like synthetic glycans. (*A*) Profiles of virus binding to v4.0 of the CFG glycan array are shown as the relative intensity of the fluorescence (RFU) for each glycan structure, indicating virus recognition and binding. The different linkage of sialylated glycans are highlighted by yellow = 2,3 SIA, blue = 2,3 and 2,6 SIA, and pink = 2,6 SIA. (*B*) The binding of each virus to human-like (6′SLN) and avian-like (3′SLN) was measured by a solid-phase binding assay. The mean and SD of five independent replicates are plotted. Statistical significance was analyzed by two-way ANOVA with Sidak’s multiple comparisons test. ****, < 0.0001; ***, < 0.001.

Reasoning that a permissive effect of N129D may occur through the interaction of the attachment and fusion functions of HA, we next evaluated HA receptor binding. The impact of HA N129D on receptor preference was assessed by comparing NL09-wt and NL09-HA-N129D viruses on a glycan array (Fig. 6*A*). Both viruses bound predominantly to Sia-α2,6 glycans. In addition, extensive overlap in bound glycans was seen, with seven of the top ten glycans common between NL09-wt and NL09-HA-N129D viruses (Fig. 6*A*). Relative differences between the viruses in binding to certain glycans suggest some change in specificity, however. With the caveat that many natural glycans present at the site of infection are not represented on the glycan arrays employed, the data suggest that N129D leads to subtle changes in receptor preference.

We then tested whether the N129D mutation modulates the strength of IAV receptor binding using a solid-phase binding assay (Fig. 6*B*). Attachment of NL09-wt virus to an avian-like synthetic Sia-α2,3 glycan (3′SLN) was at the limit of detection and the addition of N129D did not enhance this binding. In contrast, NL09-wt virus bound robustly to a human-like synthetic Sia-α2,6 glycan (6′SLN), as expected. Notably, compared to NL09-wt virus, NL09-HA-N129D virus showed higher levels of binding to 6′SLN. The short synthetic glycans used to evaluate the strength of binding are simpler and more homogenous than those found in the respiratory tract; nevertheless, our observations are consistent with published data (41) and suggest that the addition of the N129D mutation enhances the strength of HA binding to human-like receptors.

### N129D Restores Fitness of bnAb Escape Variants in Human Cell Cultures.

Owing to the clear functional relationship between HA receptor binding and membrane fusion functions during viral entry, we hypothesized that N129D acted epistatically to enable positive selection of mutations in the HA stem domain. If correct, then the fitness effects of the observed mutations in the stem domain would be reduced in the presence of N129D.

To test this concept, we first evaluated the fitness of double-mutant viruses carrying N129D in a ferret model. We focused this analysis on escape mutations with lower fitness costs, with the rationale that these mutations are more likely to support the evolution of high-fitness escape variant viruses in human circulation. In ferrets, we investigated the impact of N129D on the H8R and HA2-H111Y escape mutations and assessed both viral replication and transmission (*SI Appendix*, Fig. S9). The peak viral titers of NL09-wt virus were 10-fold higher than NL09-HA-N129D virus in the inoculated ferrets, but this impact of N129D was not observed in the viruses harboring escape mutations (*SI Appendix*, Fig. S9*A*). NL09-wt virus transmitted to two out of three recipient ferrets, with positivity first apparent at 2 or 5 day postcontact (dpc) (*SI Appendix*, Fig. S9*B*). NL09-HA-N129D transmitted to all recipients, with positivity detected uniformly at 2 day postcontact (*SI Appendix*, Fig. S9*C*). Thus, the N129D mutation may enhance airborne transmissibility of NL09 virus. The viruses harboring the individual escape mutations H8R or HA2-H111Y spread to one out of three exposed ferrets, suggesting that these mutations impose a fitness cost at the level of transmission (*SI Appendix*, Fig. S9 *D* and *F*). Likewise, viruses harboring the dual mutations, NL09-HA-H8R-N129D and NL09-HA2-H111Y-N129D, transmitted to one out of three exposed ferrets (*SI Appendix*, Fig. S9 *E* and *G*). Thus, N129D was not sufficient to overcome the deleterious effects of the escape mutations in ferrets.

**Fig. 7.**
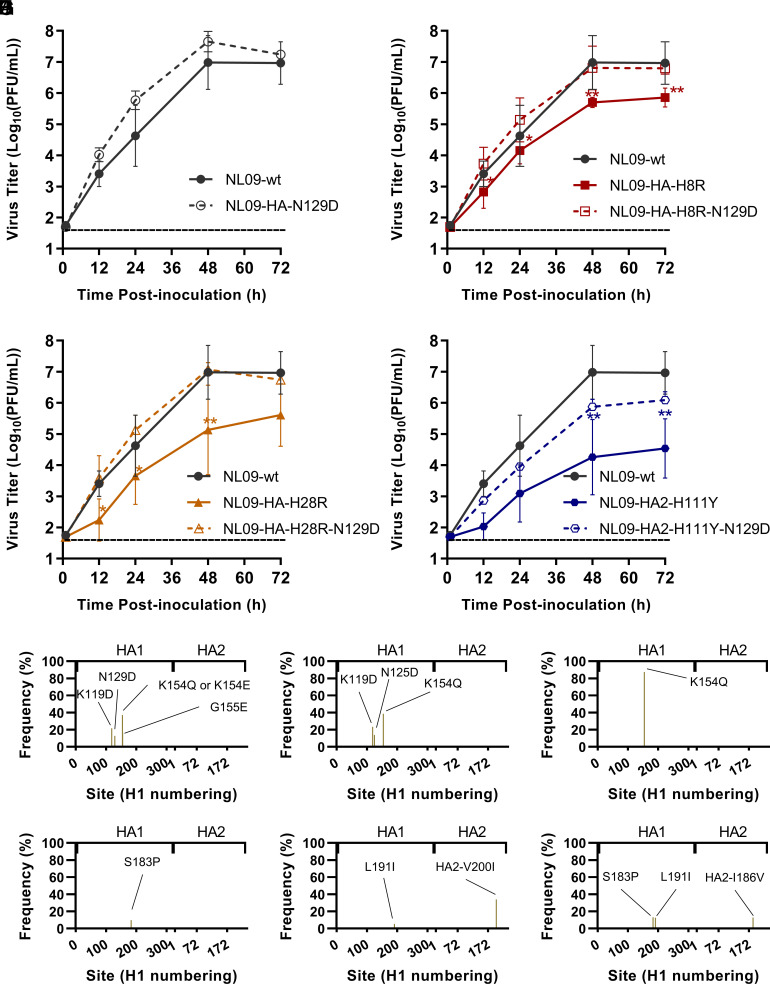
N129D mutation enhances the replication of NL09 viruses harboring individual escape mutations in NHBE cells and facilitates evolution in HA2 in the presence of a stem-bnAb. (*A*–*D*) Viral replication in NHBE cells. The mean and SD of at least three independent replicates are plotted. Statistical significance was assessed using a two-way ANOVA with Sidak’s multiple comparisons test. **, < 0.01; * < 0.05. (*E*–*G*) NL09 virus or (*H*–*J*) NL09-HA-N129D viruses were combined with 70-1F02 antibody and serially passaged four times with increasing concentration of antibody. Three replicates lineages were evaluated, with an initial concentration of 2 μg/mL.

While the outcomes observed in ferrets did not support an epistatic effect of N129D, the functional consequences of this mutation are likely to vary with the sialic acid receptors present in a given host species. We therefore examined viral fitness in primary, differentiated human bronchial epithelial cells, again focusing on the less deleterious escape mutations since they are more likely to be relevant for seasonal influenza. Recombinant viruses containing N129D and one of three individual escape mutations (NL09-HA-H8R-N129D, NL09-HA-H28R-N129D, and NL09-HA2-H111Y-N129D) were evaluated in NHBE cells (Fig. 7). The NL09-HA-N129D virus achieved higher titers than NL09-wt virus (Fig. 7*A*). Higher viral replication was also observed in the viruses carrying both mutations compared to the corresponding viruses carrying a single bnAb escape mutation. Notably, in this system, the growth of the dual mutant viruses was comparable to that of NL09-wt virus. These observations in NHBE cells clearly indicate that fitness costs incurred by escape mutations in the stem domain are mitigated by HA N129D.

Finally, to directly evaluate whether the emergence of escape mutations in the HA stem is potentiated by the preexistence of N129D, serial passaging in the presence of bnAb was performed with NL09wt and NL09-HA-N129D viruses. The two viruses were passaged in parallel four times, with increasing concentrations of 70-1F02 stem-bnAb (Fig. 7 *E*–*J*). At passage 4, we observed that several mutations arose in the HA head domain within the NL09-wt virus population. All of these head mutations are located in the vicinity of the HA antigenic site and most increased negative charge, similar to the N129D mutation. In contrast, the passage 4 population derived from NL09-HA-N129D virus did not exhibit similar mutations in the head domain but did acquire mutations in HA2. Although the observed mutations differ from those seen in the previous serial passaging experiments, the pattern of mutations in the stem arising only in the context of mutations proximal to the RBS was recapitulated, supporting a role for epistasis between the HA head and stem domains in the evolution of bnAb escape variants.

## Discussion

The HA stem domain shows high conservation among circulating IAVs, making it an attractive target for vaccination. This conservation is due at least in part to the importance of the stem region in the structure of the HA trimer and for HA-mediated membrane fusion. Here, we sought to probe the importance of functional constraints to conservation of the HA stem. We identified several escape mutations against two different stem-bnAbs, 70-1F02 and 05-2G02. All were located within the HA stem domain and abolished interaction with the antibodies; however, a range of fitness costs attributable to altered kinetics of fusion were apparent, suggesting an evolutionary trade-off between stem bnAb escape and efficient viral entry. Importantly, the fitness of bnAb escape variants in human cell cultures was restored by a mutation in the HA head that strengthened receptor interactions. Thus, in the context of immune selection acting on the functionally constrained HA stem, positive epistasis between the head and stem domains may enable antigenic evolution.

Evolutionary barriers to HA escape from immunological pressure vary with protein location and subtype. Antigenic drift in the HA stem is minimal compared to the head, consistent with reported differences in the mutational tolerance of these two domains (33, 34). In addition, compared to H3 subtype IAV, H1 subtype viruses have a higher genetic barrier to evading stem-binding antibodies (44). Our data indicate that the H1 HA stem is capable of escape from immunological pressure in exchange for reduced fitness. The escape mutations identified here show parallels with previously reported mutations that allow escape from other stem-bnAbs: H28R was observed during viral passage with a mouse monoclonal antibody, KB2 (28), while H111T or H111L allowed escape from C179 or CR6261 stem-bnAbs (15, 45). Interestingly, we observed that S54P and H111Y in HA2, located in the interior of HA trimer, escape from both 70-1F02 and 05-2G02 stem-bnAbs. Likewise, A44T or A44V in HA2, which confer escape from several stem-bnAbs, are located in the interior of the HA trimer (22, 28, 46). Mutations within this interior region are likely to alter epitope recognition through indirect effects on HA conformation, which may explain the association with escape from multiple distinct antibodies. This mode of escape is notable in that it may allow viral evasion of polyclonal responses to the stem and distinct clonotypes present in different individuals.

Comparing the frequency dynamics of variants in our passaged virus populations, we infer that changes in the head of HA (N129D or the similar N156D) typically arose prior to escape mutations. Given that N129D has negligible effects on stem-bnAb escape, this observation is perhaps counter-intuitive. Early positive selection of these two head mutations is however consistent with their occurrence in a subset of virus populations passaged without stem-bnAbs and with prior reports indicating that they affect HA receptor binding affinity (41, 43). Thus, as confirmed herein for N129D, these mutations were likely beneficial in the wild-type background.

Importantly, the evolutionary trajectories observed suggest that preoccupancy of mutations in the head domain allowed escape variants to rise to dominance. For example, in one lineage, the escape mutation HA2-A44V arose in early passages in the absence of any changes in the head domain, but then was lost from the population by passage 10. Conversely, the H8R mutation that arose within the same lineage reached high frequencies in the context of the N129D mutation. Coexistence of HA head and stem mutations has been previously observed in the context of viral passage with stem-binding antibodies (26, 28, 29, 32, 47); however, these changes were attributed to cell culture adaptation and not thoroughly evaluated for epistasis with the stem mutations. Importantly, our findings suggest that these changes in the head domain were important for enabling changes in the stem. The deleterious side effects of antigenic changes constrain adaptation by preventing positive selection of antigenic variants. Fitness effects are context-dependent; however, such that an antigenic change may not incur costs if it occurs within the background of a permissive variant (48–50). Notably, the addition of N129D to recombinant viruses harboring individual escape mutations enhanced replication in human cell cultures, yielding phenotypes comparable to that of NL09-wt virus. This pattern mirrors that seen in the evolution of oseltamivir resistance in the human seasonal H1N1 lineage in which permissive mutations in NA enabled the acquisition of resistance mutations that typically incurred fitness costs (51). We therefore propose that the N129D mutation acts as a permissive mutation in the context of antibody escape. The recent sweep of HA N129D in pandemic H1N1 lineage IAVs circulating globally may therefore have important implications for further viral evolution.

The head and stem domains of HA perform distinct functions during viral entry, raising the question of how mutation in the head could modulate the fitness effects of mutation in the stem. The observed increase in viral receptor binding with the introduction of HA N129D suggests the following model. The prefusion conformation of HA proteins carrying bnAb escape mutations in the stem domain is destabilized, such that unfolding to the postfusion conformation is more likely to occur prematurely (18, 31, 45). This destabilization may in turn increase the fraction of HA protomers on a virion that are unable to mediate receptor binding, lowering the avidity of virion binding to cells. The consequences of this defect for viral attachment would be expected to depend on the affinity of remaining prefusion HA protomers for sialic acid receptors. The fitness effects of destabilizing mutations in the stem may therefore be modulated by amino acids proximal to the RBS. While our data are consistent with this model, it presents additional hypotheses that should be tested in future studies. Notably, mutation in NA that modulates its enzymatic activity was previously shown to contribute to IAV escape from stem-bnAbs (26). Although this change in NA alone increased resistance to neutralization, it may also have had a permissive effect by modulating the balance between HA and NA functions in viral attachment and release (26).

In one lineage passaged in the presence of bnAb, we observed N11T and T270A mutations at high frequency. Both lie in regions of HA1 that are within the stem domain. Surprisingly, these changes did not affect sensitivity to neutralization in the context of recombinant viruses. N11T removes a glycosylation site; in turn, this change is expected to improve the accessibility of the cleavage loop to proteases, resulting in more efficient HA cleavage activation (37, 38). It is possible that mutations that alter cleavage of HA0 to HA1 and HA2 modulate constraints on HA conformation stability, in a like manner to mutations that affect receptor binding. In particular, if increased efficiency of cleavage increases the fraction of HA protomers competent for entry, this could offset a reduction in functional protomers brought about by destabilizing mutations in the HA stem. Alternatively, decreased efficiency of cleavage may reduce the opportunity for premature adoption of the postfusion conformation since cleavage activation is a prerequisite of this conformational change. The apparent positive selection of T270A may relate to its positioning near a disulfide bridge that links the ascending and descending chains of HA1 (52). Although outside the scope of the present study focused on bnAb escape, the fitness implications of these two HA mutations warrants further investigation.

Despite the beneficial effects of N129D in primary cell cultures and its positive selection in humans, this mutation did not show a permissive phenotype in ferrets. As expected, viruses carrying escape mutations in the HA stem domain transmitted poorly in ferrets. However, coupling of these escape mutations to N129D did not result in the enhancement of transmission, as was expected based on our observations in primary human cells. The use of ferrets to model these processes presents a limitation, however. Although shown to be similar in prevalence of α2,3 and α2,6 linked sialylated glycans (53–55), the distribution of specific glycan species likely differs between ferret and human respiratory tracts. We hypothesize that these differences underlie the differing effects of N129D in the two hosts. While a valuable model for examining many viral processes, we caution that the ferret system—or any animal model—cannot fully recapitulate the evolutionary pressures relevant in humans.

In summary, our data suggest that evolution of circulating IAV in response to increased selective pressure on the HA stem domain would likely be highly constrained. However, epistasis may increase the potential for the emergence of variants that escape neutralization by stem-bnAbs. Therefore, active surveillance to monitor viral antigenic evolution will be needed along with universal vaccine development.

## Materials and Methods

### Cells, Viruses, and Antibodies.

MDCK cells were maintained in minimal essential medium (MEM) (Gibco, USA) supplemented with 10% fetal bovine serum (FBS) (R&D SYSTEMS, USA) and penicillin-streptomycin (PS) (Gibco, USA). Baby Hamster Kidney (BHK-21) cells were maintained in MEM with GlutaMAX™ Supplement (Gibco, USA), 5% FBS and PS. 293T cells were maintained in Dulbecco's Modified Eagle Medium (Gibco, USA) supplemented with 10% FBS and PS. NHBE cells (Lonza, Switzerland) were amplified and differentiated into air–liquid interface cultures as recommended by Lonza and described previously (56). Influenza A/Netherlands/602/2009 (H1N1) [NL09] virus was generated using reverse genetics. Briefly, 293T cells transfected with reverse genetics plasmids 24 h previously were cocultured with MDCK cells at 37 °C for 48 h. Recovered virus was plaque purified and propagated in MDCK cells to generate a working stock. 70-1F02 and 1009-3E04 antibodies were isolated from patients who were naturally infected with 2009 pandemic H1N1 virus, and 05-2G02 antibody was isolated from an immunized individual (20, 24).

### Selection of Escape Mutants.

To isolate escape variants, NL09 virus was serially passaged 10 times with a range of the stem bnAbs. First, single replicates with an initial concentration of 20, 2, or 1 µg/mL of 70-1F02 or 05-2G02 were mixed with an MOI = 1 PFU/cell of NL09 virus for 1 h at 37 °C. Next, the mixtures were inoculated onto a 6-well plate of MDCK cells and incubated for 2 d at 37 °C. The supernatant was collected, mixed with increased concentration of the stem-bnAbs, and inoculated onto fresh MDCK cells. During serial passage with the stem-bnAbs, the antibody concentration was increased twofold if the infected cells showed gross cytopathic effects (CPE) (70 to 90% floating cells) (35). If CPE was moderate to mild, the antibody concentration was maintained. As a control, viruses without stem-bnAbs were passaged in parallel in MDCK cells. The populations passaged 10 times and the early passaged populations which showed moderate resistance to stem-bnAb were deep-sequenced to monitor variant dynamics. In addition, six plaques were picked from the passaged populations and sequenced to verify the deep sequencing results and to evaluate the cooccurrence of mutations (*SI Appendix*, Fig. S2 and Datasets S1–S3).

### Next-Generation Sequencing and Variant Analysis.

Analysis of nonconsensus variants was made using LoFreq (57) following the Genome Analysis Toolkit best practices (58). In brief, after removing adapters using Trimmomatic (version 0.39) (59), reads were aligned to their reference sequence using the option mem from BWA (60). Data formatting for GATK was made using Picard (http://broadinstitute.github.io/picard/). Reads were realigned using RealignerTargetCreator and IndelRealigner from GATK. The quality of bases was recalculated using BaseRecalibrator from GATK. The resulting bam file was used to perform variant calling analysis by LoFreq. Only variants at a frequency of 0.05 with a coverage equal or above 400 were used. Amino acid positions are indicated with H1 numbering, in which position 1 is designated as aspartic acid following the N-terminal signal peptide (MKAILVVLLYTFATANA) (61).

### Site-Directed Mutagenesis and Recombinant Virus Rescue.

All viruses used in this study were generated by reverse genetics (62). First, 293T cells were transfected with eight bidirectional plasmids encoding PB2, PB1, PA, HA, NA, NP, M, and NS genes. After 24 h posttransfection, 1 × 10^6^ cells of MDCK cells were overlaid with virus media containing 1 µg/mL TPCK-treated trypsin. Rescued viruses were plaque purified and passaged once in MDCK cells. The resultant cell passage 1 stocks were used in experiments. Single amino acid mutations were introduced into the NL09 HA by site-directed mutagenesis using primers (*SI Appendix*, Table S1).

### Plaque Reduction Neutralization Test.

The diluted 70-1F02 or 05-2G02 antibodies were mixed 1:1 with 100 PFU of viruses and incubated for 1 h at 37 °C. After 1 h, the fully confluent MDCK cells were inoculated with the antibody-virus mixture and incubated for 1 h at 37 °C. After washing with PBS, the cells were overlaid with 2× MEM with 1 % Oxoid agar (Thermo Fisher Scientific, USA) containing 1 µg/mL TPCK-treated trypsin. After 2 d of incubation at 37 °C, the plaques were counted, and the concentration of antibodies leading to a 50% reduction in plaque counts (PRNT_50_ titer) was calculated.

### Syncytia Formation Assay.

BHK-21 cells were transfected with 1 µg of pCAGGS plasmid encoding the NL09 wild-type or escape mutant HA proteins using Lipofectamine (Invitrogen, USA) and Plus reagent (Invitrogen, USA). At 24 h posttransfection, HA-expressing cells were washed once with PBS and treated with 2.5 µg/mL of TPCK-treated trypsin (Sigma-Aldrich, USA) for 10 min. Afterward, the cells were exposed to pH adjusted PBS for 5 min at 37 °C. The cells were neutralized by complete growth media (MEM, supplemented with GlutaMAX™ Supplement, 5% FBS and PS) and incubated for 2 h at 37 °C to allow syncytia formation. The cells were fixed and stained with the Hema3Stat Pak (Fisher Scientific, USA) according to the manufacturer’s protocol. Syncytia were visualized and photographed using a Zeiss Axio Observer inverted microscope with an attached digital camera (Zeiss, Germany).

### Cell-Based ELISA.

BHK-21 cells in a 96-well plate were transfected with 100 ng of pCAGGS NL09 HA (wild-type or escape mutants) using Lipofectamine 3000 (Invitrogen, USA). At 24 h posttransfection, the HA-expressing cells were washed twice with PBS and fixed with 4% formaldehyde for 2 h. The fixed cells were washed twice with PBS and blocked with 5% skim milk in PBS with 0.05% Tween20 for 1 h. Serially diluted stem-bnAbs were added on to the plate and incubated for 1 h at room temperature and then washed 4 times with PBST (PBS + 0.05% Tween20). The secondary antibody [anti-human IgG (H+L)-HRP conjugated, Promega, USA] was added to the plate and incubated for 1 h. After washing with PBST four times, the plates were treated with 100 µL of TMB solution (Thermo Fish Scientific, USA) and incubated for 15 min. The reactions were halted by addition of 2 N sulfuric acid. The color was read at 450 nm with the reference wavelength of 655 nm.

### Viral Fusion Kinetics.

MDCK cells in a 96-well plate were infected at an MOI = 1 PFU/cell. At 24 h postinfection, the cells were washed twice with PBS and treated with 5 µg/mL TPCK-treated trypsin for 10 min at 37 °C. The trypsin was neutralized by complete growth media (MEM supplemented with 10% FBS and PS). After washing with PBS, the cells were incubated with 1 µg/mL of 1009-3E04 antibody for 1 h at 37 °C. Following the antibody treatment, the cells were washed twice with PBS containing 2% FBS (PBSF) and then treated with pH-adjusted PBS at pH = 5.0 for 0, 2, 4, 6, 8, and 10 min at 37 °C. Following the acid treatment, the cells were washed with PBSF and then incubated with anti-human IgG-HRP for 1 h at room temperature. After washing with PBSF, the cells were treated with 100 µL of TMB solution and incubated for 15 min. The reactions were halted by addition of 2 N sulfuric acid. The color was read at 450 nm with the reference wavelength of 655 nm.

### Multistep Viral Growth Kinetics in MDCK, A549, and NHBE Cells.

MDCK and A549 cells were seeded onto a 6-well plate. After 24 h incubation, the cells were washed three times with PBS and inoculated with MOI 0.002 PFU/cell of NL09 wild-type or NL09 escape mutant viruses for MDCK cells and MOI 0.01 PFU/cell for A549 cells. After 1 h absorption at 4 °C, the cells were washed three times with PBS and virus media with 1 µg/mL TPCK-treated trypsin (Sigma-Aldrich, USA) was added. A 200 µL volume of supernatant was harvested at each time-point with replacement of the same volume of virus medium. NHBE cells were differentiated at an air–liquid interface. After the mucus was removed from the apical surface, the cells were washed five times with PBS and inoculated with MOI 0.01 PFU/cell of NL09 wild-type or NL09 escape mutant viruses.

### Solid Phase Binding Assay.

Serially diluted (20, 10, 5, 2.5, 1.25, 0.625, and 0.3125 µg/mL) biotinylated glycan 3′SLN-PAA and 6′SLN-PAA (Carbosynth, USA) in PBS were added to the wells of streptavidin-coated high binding capacity 96-well plates (Thermo Fisher Scientific, USA). After incubation overnight at 4 °C, the plates were washed with PBST, blocked with PBS containing 2% Bovine serum albumin (BSA) for 2 h at room temperature, and then washed with PBST. Then, 10^6^ PFU of virus was prepared in PBS containing 10 µM GS4071 neuraminidase inhibitor and added to the wells and incubated overnight at 4 °C. The plates were washed with PBST and incubated with 5 µg/mL 1009-3E04 monoclonal antibody for an hour. After washing with PBST, the wells were incubated with secondary antibody [anti-human IgG (H+L)-HRP conjugated, Promega, USA] for an hour and then washed with PBST again. The plates were treated with 100 µL of TMB solution and incubated for 15 min. The reactions were halted by addition of 2 N sulfuric acid. The color was read at 450 nm with the reference wavelength of 655 nm.

### Glycan Microarray.

The viruses were purified through a 25% sucrose cushion, then labeled with Alexa Fluor 488 (Invitrogen, USA) as previously described (63, 64). Labeled viruses were dialyzed against PBS using a 7,000-molecular-weight-cutoff Slide-A-Lyzer mini-dialysis unit (Thermo Fisher Scientific, USA) overnight at 4 °C. The labeled viruses were run on v4.0 of the CFG Glycan Array using the buffers and conditions described previously (63, 64).

### Ferret Transmission Experiment.

Ferret studies were approved and conducted in compliance with all the regulations stated by the Institutional Animal Care and Use Committee (IACUC) of the University of Georgia (AUP2019 03-021-Y3-A7). Studies were conducted under BSL-2 conditions at the Poultry Diagnostic and Research Center, University of Georgia. Animal studies and procedures were performed according to the IACUC Guidebook of the Office of Laboratory Animal Welfare and PHS Policy on Humane Care and Use of Laboratory Animals. Animal studies were carried out in compliance with the ARRIVE guidelines (https://arriveguidelines.org). Twenty-week-old female ferrets were acquired from Triple F Farms (Gillett, PA, USA). The next day after arrival, ferrets were anesthetized with an i.m. injection (0.5 mL/kg) of a ketamine cocktail (20 mg/kg Ketamine, 1 mg/kg Xylazine) in the thighs, followed by blood collection to test serum samples for prior IAV infection using NP ELISA (IDEXX, ME, USA). All ferrets tested negative for prior IAV exposure. A subcutaneous implantable temperature transponder (BMDS, DE, USA) was inserted in the back of the neck to ID and monitor body temperature of each ferret. Ferrets were acclimated for 7 d upon arrival before virus challenge. For each virus evaluated, ferrets (n = 3) were anesthetized as described above and inoculated with 1 × 10^5^ TCID_50_/ferret in a final volume of 1 mL in 1× PBS. On day 1 postinoculation (dpi), the direct inoculated ferrets were placed with naive respiratory contact ferrets (ratio 1:1/isolator). Temperature, weight, and clinical signs were monitored every day. Nasal wash samples were obtained for all the ferrets every day until 9 dpi (8 d postcontact, dpc) by introducing 1 mL volume of 1× PBS-BSA 1% into the nasal passages to induce sneezing, collecting the aspirate using a sterile plastic dish, and washing the plate with an additional 1 mL of 1× PBS supplemented with antibiotic/antimycotic solution (Gibco, USA). Nasal washes were then aliquoted and frozen at −80 °C until further processing. To avoid direct-contact contamination, 70% EtOH surface decontamination of gloves and equipment was performed between handling inoculated and contact animals. Contact animals were always handled before inoculated animals. Different bite-resistant gloves, utensils, and tools were used between contact and virus-inoculated animals, with disposable gowns and gloves changed between isolators. On 14 dpi, all ferrets were anesthetized as described above, bled, and humanely euthanized using 1 mL of Euthasol® (Virbac, TX, USA).

### Sequence Analysis.

3341 HA sequences of 2009 pandemic H1N1 virus isolates from 2009 to 2021 were collected in GISAID (Global Initiative on Sharing All Influenza Data) on September 25th, 2021 and sorted by years in Excel.

## Supplementary Material

Appendix 01 (PPTX)

Dataset 01 (XLSX)

Dataset 02 (XLSX)

Dataset 03 (XLSX)

Dataset 04 (XLSX)

Dataset 05 (XLSX)

Dataset 06 (XLSX)

Dataset 07 (XLSX)

Dataset 08 (XLSX)

Dataset 09 (XLSX)

Dataset 10 (XLSX)

Dataset 11 (XLSX)

Dataset 12 (XLSX)

Dataset 13 (XLSX)

Dataset 14 (XLSX)

Dataset 15 (XLSX)

Dataset 16 (XLSX)

Dataset 17 (XLSX)

Dataset 18 (XLSX)

## Data Availability

Deep sequencing data are available from the Sequence Read Archive under BioProject Accession PRJNA842182. Sequences determined from plaque isolates are included as Datasets S1–S3. Raw data used for the generation of Figs. 1–7 and *SI Appendix*, Figs. S1–S9 are included as Datasets S4–S18.
