# Supplementary material for: Epistasis reduces fitness costs of influenza A virus escape from stem-binding antibodies
Source: Proc Natl Acad Sci U S A. 2023 Apr 17;120(17):e2208718120. doi: 10.1073/pnas.2208718120 (PMC10151473; doi:10.1073/pnas.2208718120)
Supplement: Supplementary file 1 — Appendix 01 (PPTX) [file pnas.2208718120.sapp.pptx]

## Slide 1
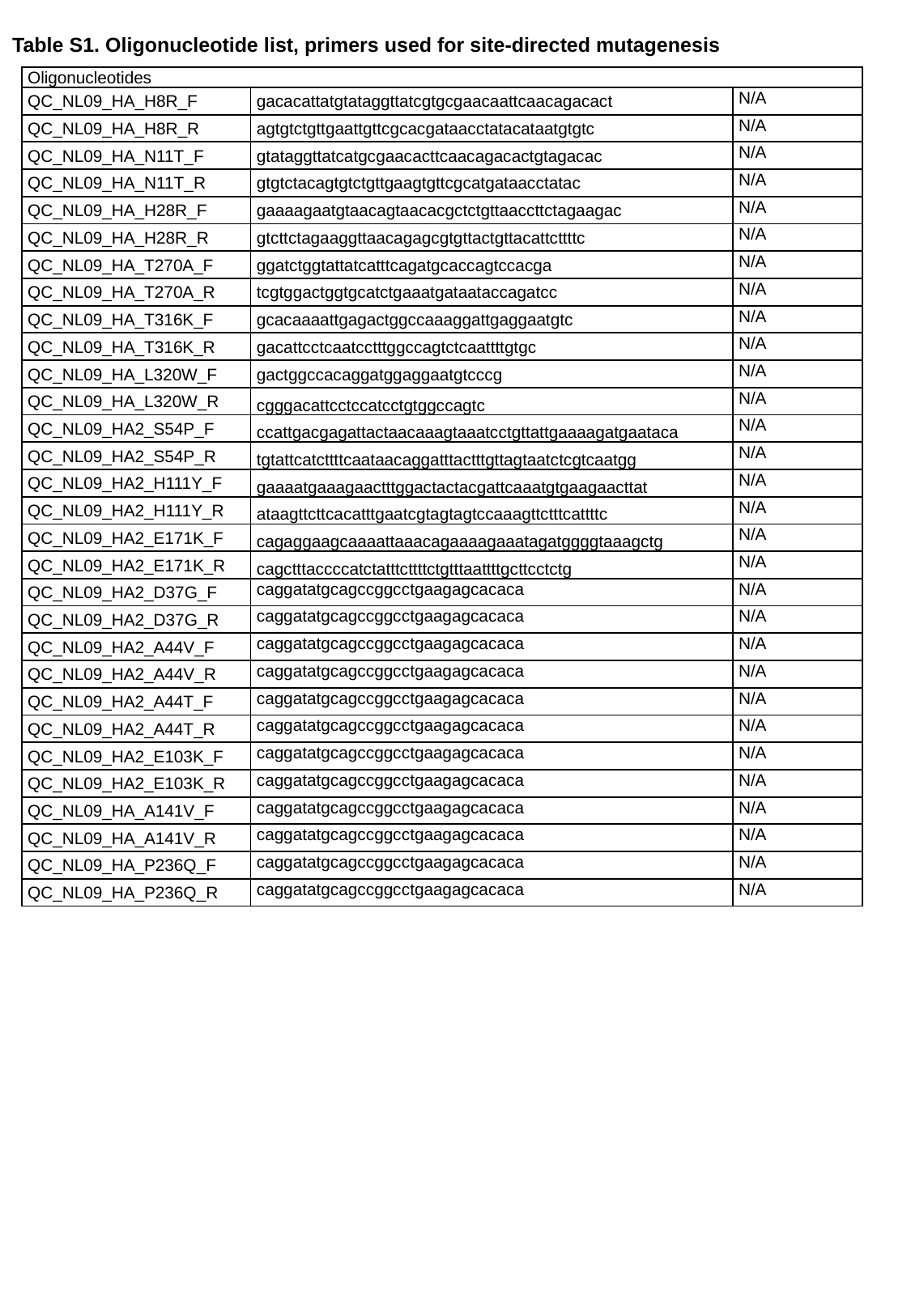

Table S1. Oligonucleotide list, primers used for site-directed mutagenesis
| Oligonucleotides | | |
| --- | --- | --- |
| QC\_NL09\_HA\_H8R\_F | gacacattatgtataggttatcgtgcgaacaattcaacagacact | N/A |
| QC\_NL09\_HA\_H8R\_R | agtgtctgttgaattgttcgcacgataacctatacataatgtgtc | N/A |
| QC\_NL09\_HA\_N11T\_F | gtataggttatcatgcgaacacttcaacagacactgtagacac | N/A |
| QC\_NL09\_HA\_N11T\_R | gtgtctacagtgtctgttgaagtgttcgcatgataacctatac | N/A |
| QC\_NL09\_HA\_H28R\_F | gaaaagaatgtaacagtaacacgctctgttaaccttctagaagac | N/A |
| QC\_NL09\_HA\_H28R\_R | gtcttctagaaggttaacagagcgtgttactgttacattcttttc | N/A |
| QC\_NL09\_HA\_T270A\_F | ggatctggtattatcatttcagatgcaccagtccacga | N/A |
| QC\_NL09\_HA\_T270A\_R | tcgtggactggtgcatctgaaatgataataccagatcc | N/A |
| QC\_NL09\_HA\_T316K\_F | gcacaaaattgagactggccaaaggattgaggaatgtc | N/A |
| QC\_NL09\_HA\_T316K\_R | gacattcctcaatcctttggccagtctcaattttgtgc | N/A |
| QC\_NL09\_HA\_L320W\_F | gactggccacaggatggaggaatgtcccg | N/A |
| QC\_NL09\_HA\_L320W\_R | cgggacattcctccatcctgtggccagtc | N/A |
| QC\_NL09\_HA2\_S54P\_F | ccattgacgagattactaacaaagtaaatcctgttattgaaaagatgaataca | N/A |
| QC\_NL09\_HA2\_S54P\_R | tgtattcatcttttcaataacaggatttactttgttagtaatctcgtcaatgg | N/A |
| QC\_NL09\_HA2\_H111Y\_F | gaaaatgaaagaactttggactactacgattcaaatgtgaagaacttat | N/A |
| QC\_NL09\_HA2\_H111Y\_R | ataagttcttcacatttgaatcgtagtagtccaaagttctttcattttc | N/A |
| QC\_NL09\_HA2\_E171K\_F | cagaggaagcaaaattaaacagaaaagaaatagatggggtaaagctg | N/A |
| QC\_NL09\_HA2\_E171K\_R | cagctttaccccatctatttcttttctgtttaattttgcttcctctg | N/A |
| QC\_NL09\_HA2\_D37G\_F | caggatatgcagccggcctgaagagcacaca | N/A |
| QC\_NL09\_HA2\_D37G\_R | caggatatgcagccggcctgaagagcacaca | N/A |
| QC\_NL09\_HA2\_A44V\_F | caggatatgcagccggcctgaagagcacaca | N/A |
| QC\_NL09\_HA2\_A44V\_R | caggatatgcagccggcctgaagagcacaca | N/A |
| QC\_NL09\_HA2\_A44T\_F | caggatatgcagccggcctgaagagcacaca | N/A |
| QC\_NL09\_HA2\_A44T\_R | caggatatgcagccggcctgaagagcacaca | N/A |
| QC\_NL09\_HA2\_E103K\_F | caggatatgcagccggcctgaagagcacaca | N/A |
| QC\_NL09\_HA2\_E103K\_R | caggatatgcagccggcctgaagagcacaca | N/A |
| QC\_NL09\_HA\_A141V\_F | caggatatgcagccggcctgaagagcacaca | N/A |
| QC\_NL09\_HA\_A141V\_R | caggatatgcagccggcctgaagagcacaca | N/A |
| QC\_NL09\_HA\_P236Q\_F | caggatatgcagccggcctgaagagcacaca | N/A |
| QC\_NL09\_HA\_P236Q\_R | caggatatgcagccggcctgaagagcacaca | N/A |

## Slide 2
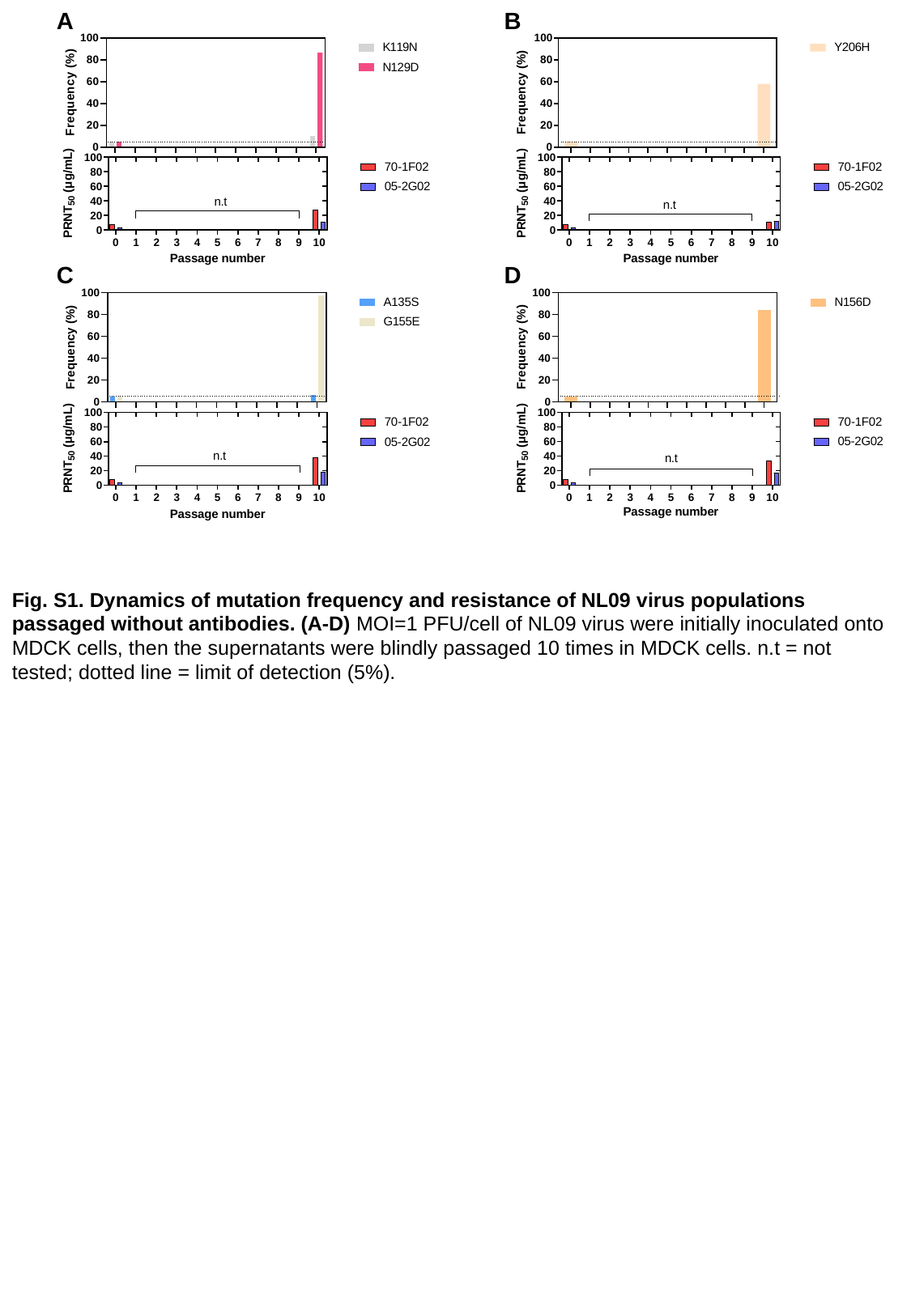

B
A
D
C
Fig. S1. Dynamics of mutation frequency and resistance of NL09 virus populations passaged without antibodies. (A-D) MOI=1 PFU/cell of NL09 virus were initially inoculated onto MDCK cells, then the supernatants were blindly passaged 10 times in MDCK cells. n.t = not tested; dotted line = limit of detection (5%).

## Slide 3
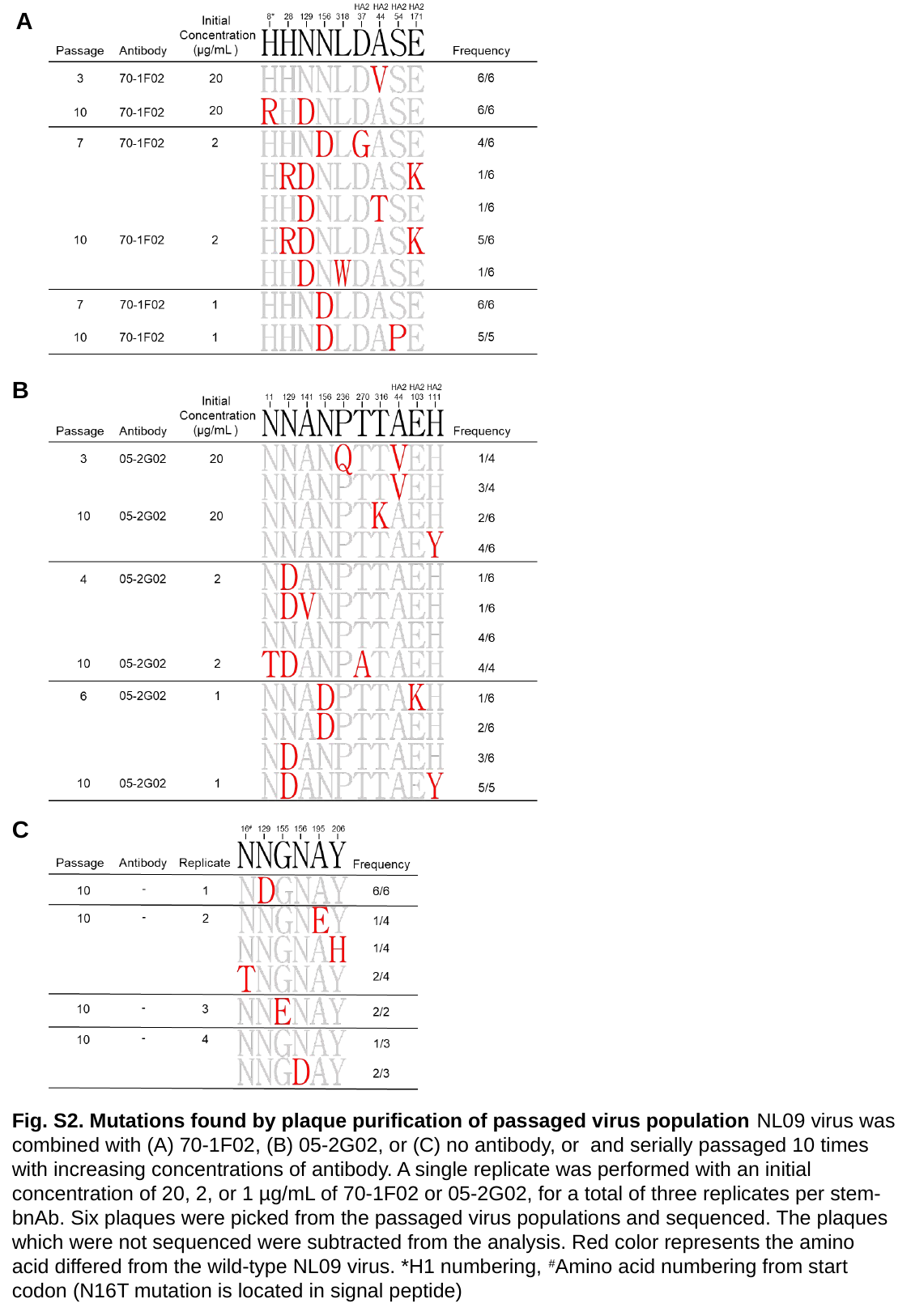

A
B
C
Fig. S2. Mutations found by plaque purification of passaged virus population NL09 virus was combined with (A) 70-1F02, (B) 05-2G02, or (C) no antibody, or and serially passaged 10 times with increasing concentrations of antibody. A single replicate was performed with an initial concentration of 20, 2, or 1 µg/mL of 70-1F02 or 05-2G02, for a total of three replicates per stem-bnAb. Six plaques were picked from the passaged virus populations and sequenced. The plaques which were not sequenced were subtracted from the analysis. Red color represents the amino acid differed from the wild-type NL09 virus. *H1 numbering, #Amino acid numbering from start codon (N16T mutation is located in signal peptide)

## Slide 4
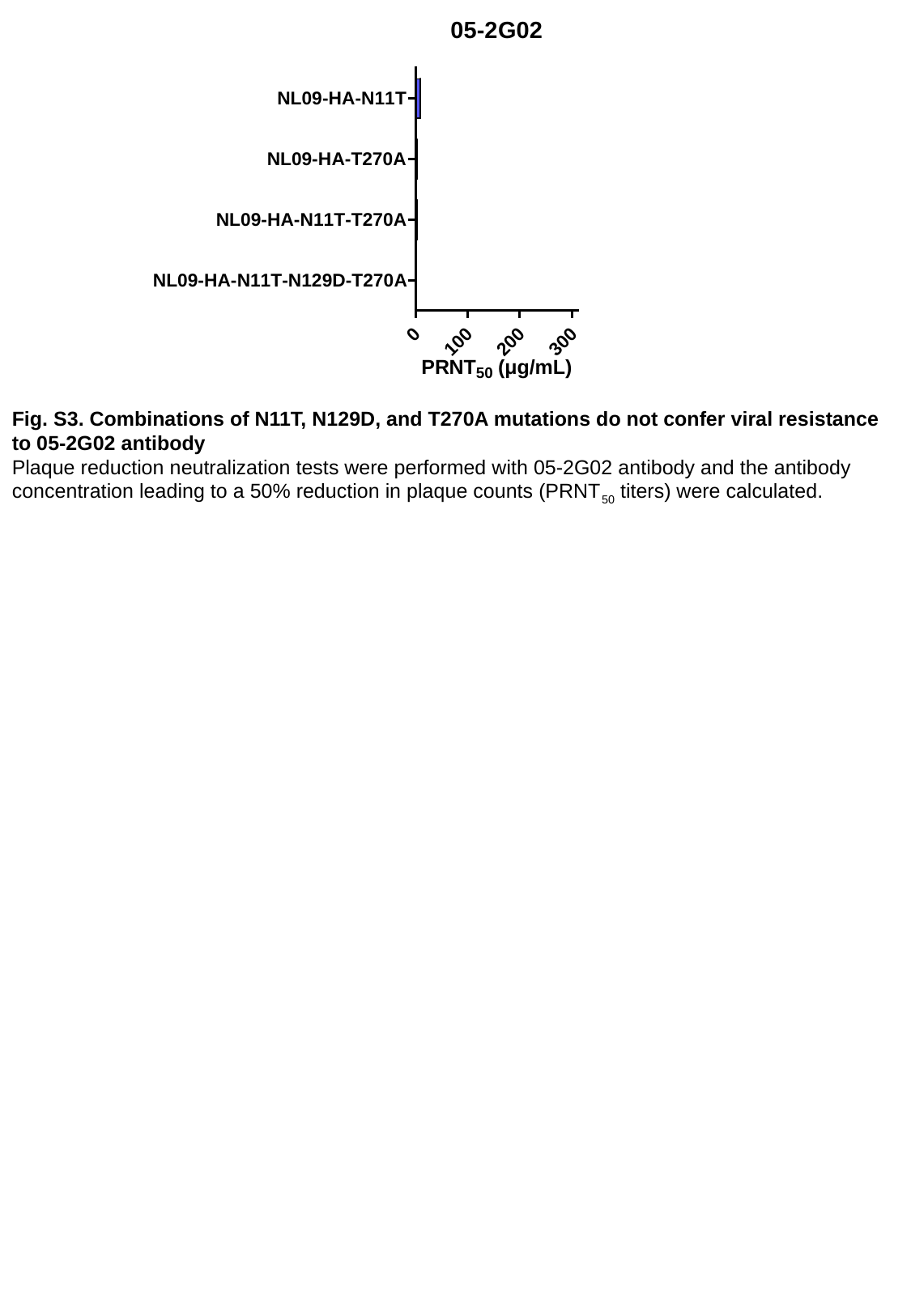

Fig. S3. Combinations of N11T, N129D, and T270A mutations do not confer viral resistance to 05-2G02 antibody
Plaque reduction neutralization tests were performed with 05-2G02 antibody and the antibody concentration leading to a 50% reduction in plaque counts (PRNT50 titers) were calculated.

## Slide 5
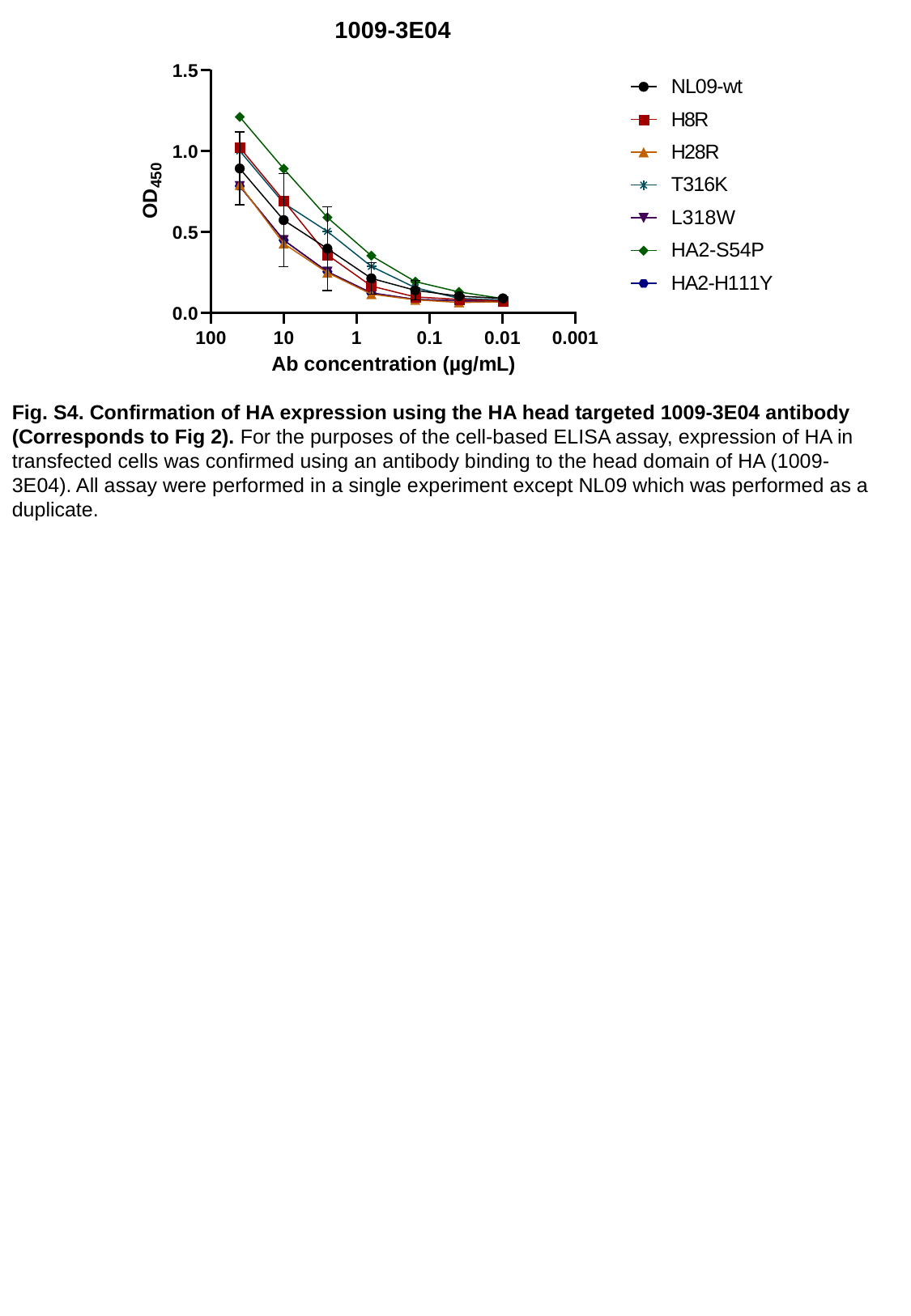

Fig. S4. Confirmation of HA expression using the HA head targeted 1009-3E04 antibody (Corresponds to Fig 2). For the purposes of the cell-based ELISA assay, expression of HA in transfected cells was confirmed using an antibody binding to the head domain of HA (1009-3E04). All assay were performed in a single experiment except NL09 which was performed as a duplicate.

## Slide 6
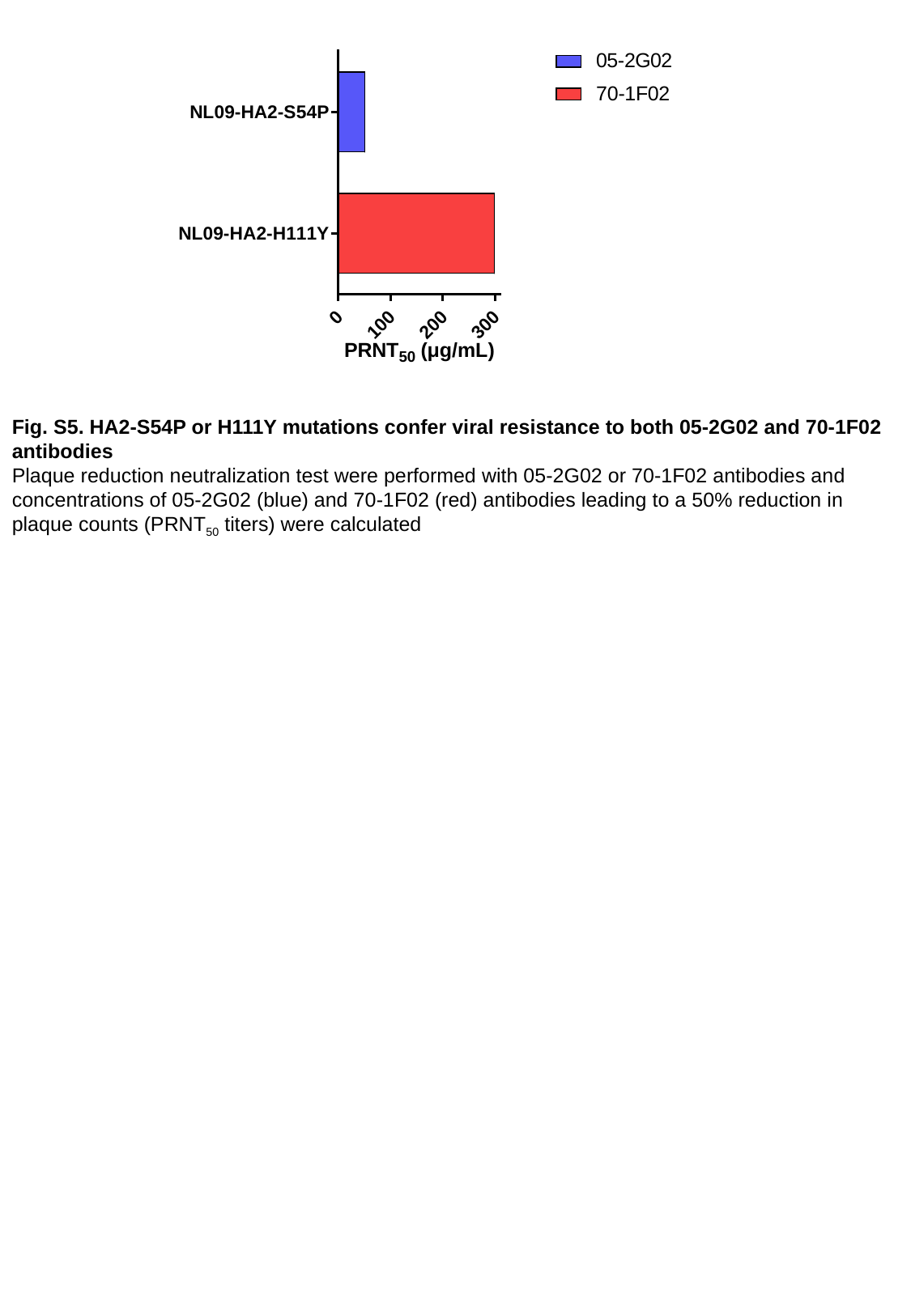

Fig. S5. HA2-S54P or H111Y mutations confer viral resistance to both 05-2G02 and 70-1F02 antibodies
Plaque reduction neutralization test were performed with 05-2G02 or 70-1F02 antibodies and concentrations of 05-2G02 (blue) and 70-1F02 (red) antibodies leading to a 50% reduction in plaque counts (PRNT50 titers) were calculated

## Slide 7
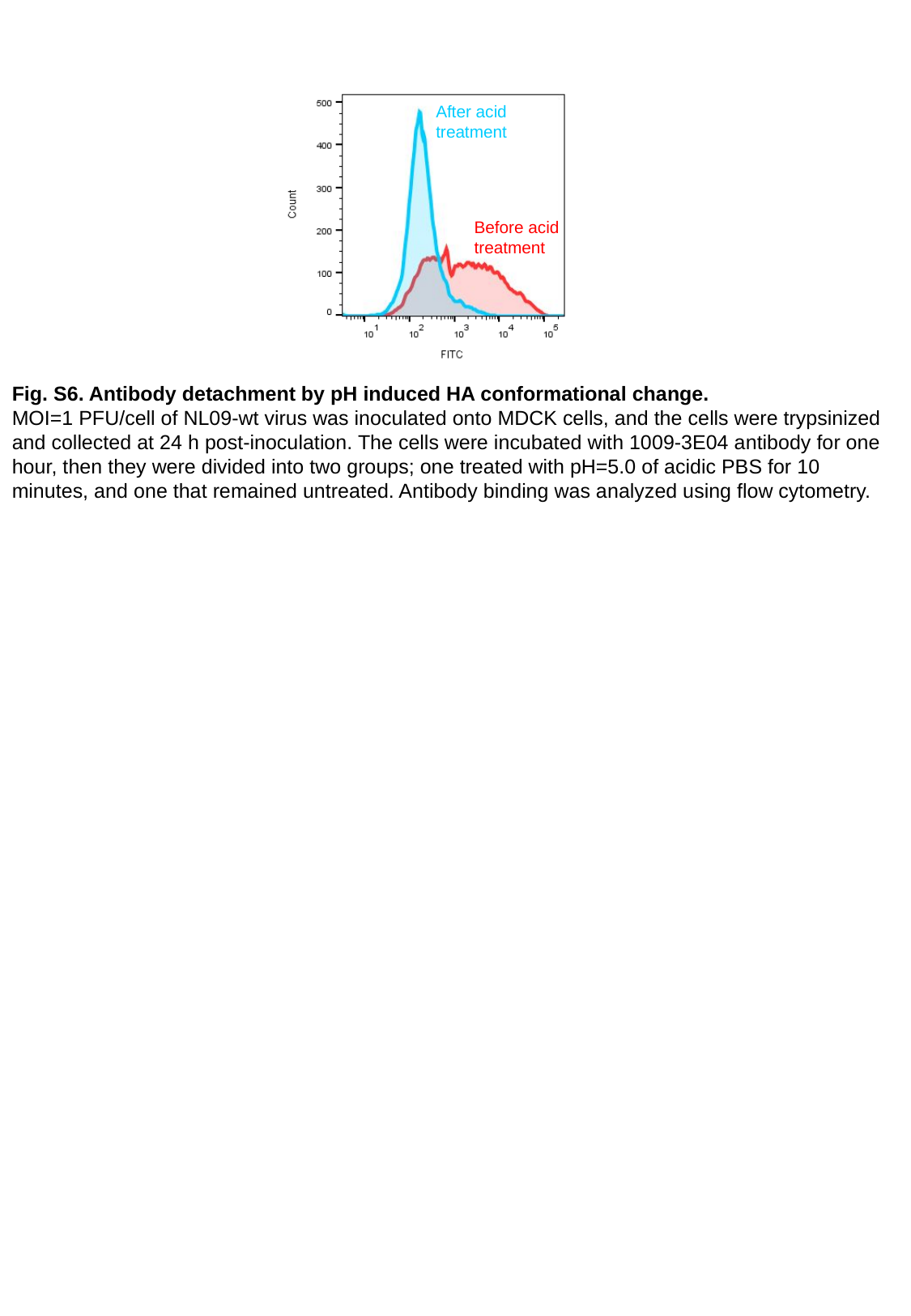

After acid treatment
Before acid treatment
Fig. S6. Antibody detachment by pH induced HA conformational change.
MOI=1 PFU/cell of NL09-wt virus was inoculated onto MDCK cells, and the cells were trypsinized and collected at 24 h post-inoculation. The cells were incubated with 1009-3E04 antibody for one hour, then they were divided into two groups; one treated with pH=5.0 of acidic PBS for 10 minutes, and one that remained untreated. Antibody binding was analyzed using flow cytometry.

## Slide 8
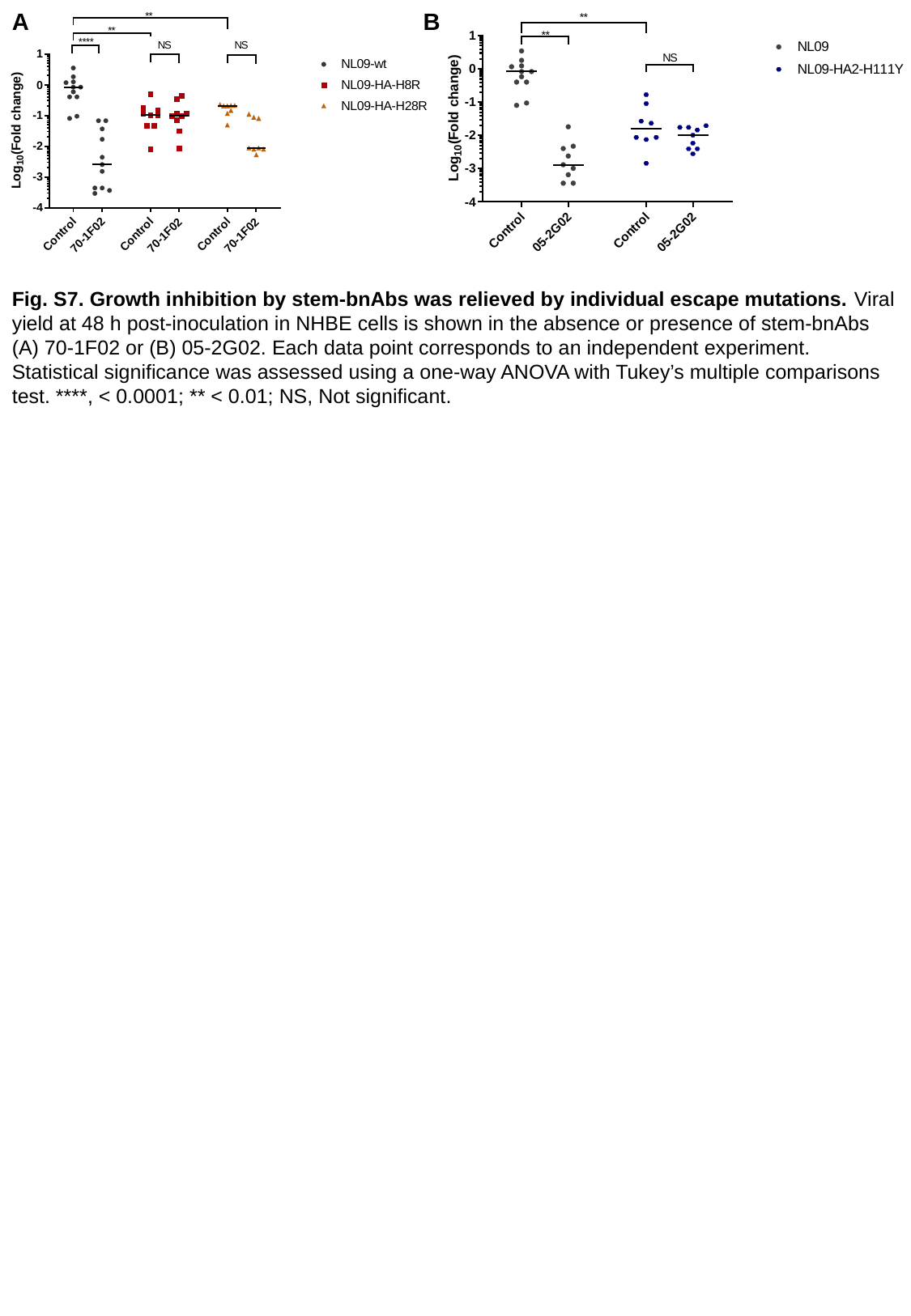

A
B
Fig. S7. Growth inhibition by stem-bnAbs was relieved by individual escape mutations. Viral yield at 48 h post-inoculation in NHBE cells is shown in the absence or presence of stem-bnAbs (A) 70-1F02 or (B) 05-2G02. Each data point corresponds to an independent experiment. Statistical significance was assessed using a one-way ANOVA with Tukey’s multiple comparisons test. ****, < 0.0001; ** < 0.01; NS, Not significant.

## Slide 9
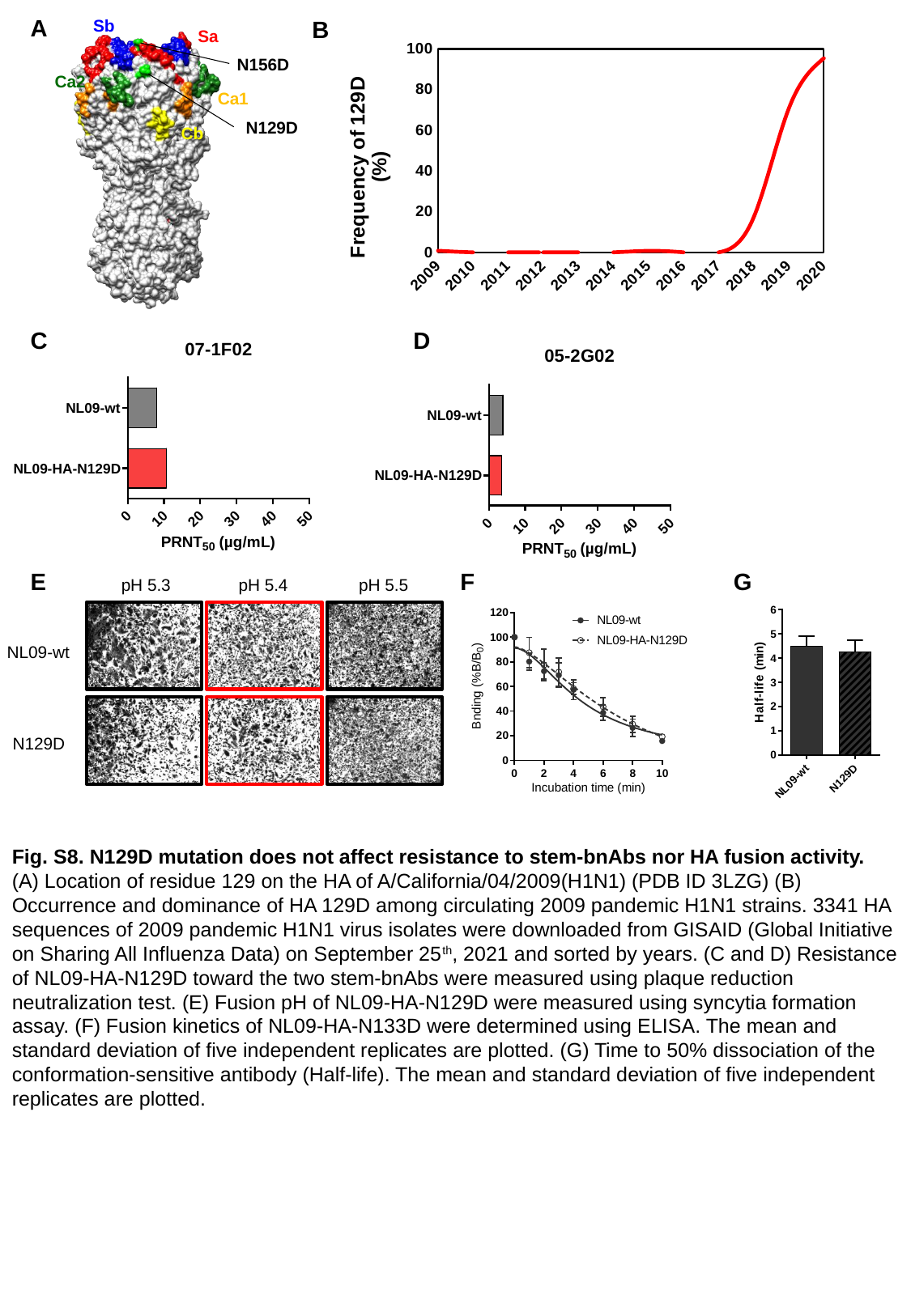

A
Sb
B
Sa
### Chart
| Category | |
|---|---|
| 2009 | 0.7614213197969544 |
| 2010 | 0.0 |
| 2011 | 0.0 |
| 2012 | 0.0 |
| 2013 | 0.0 |
| 2014 | 0.0 |
| 2015 | 0.7092198581560284 |
| 2016 | 0.0 |
| 2017 | 0.0 |
| 2018 | 17.16937354988399 |
| 2019 | 70.64116985376828 |
| 2020 | 95.38784067085953 |N156D
Ca2
Ca1
N129D
Cb
C
D
F
G
E
pH 5.5
pH 5.4
pH 5.3
NL09-wt
N129D
Fig. S8. N129D mutation does not affect resistance to stem-bnAbs nor HA fusion activity.
(A) Location of residue 129 on the HA of A/California/04/2009(H1N1) (PDB ID 3LZG) (B) Occurrence and dominance of HA 129D among circulating 2009 pandemic H1N1 strains. 3341 HA sequences of 2009 pandemic H1N1 virus isolates were downloaded from GISAID (Global Initiative on Sharing All Influenza Data) on September 25th, 2021 and sorted by years. (C and D) Resistance of NL09-HA-N129D toward the two stem-bnAbs were measured using plaque reduction neutralization test. (E) Fusion pH of NL09-HA-N129D were measured using syncytia formation assay. (F) Fusion kinetics of NL09-HA-N133D were determined using ELISA. The mean and standard deviation of five independent replicates are plotted. (G) Time to 50% dissociation of the conformation-sensitive antibody (Half-life). The mean and standard deviation of five independent replicates are plotted.

## Slide 10
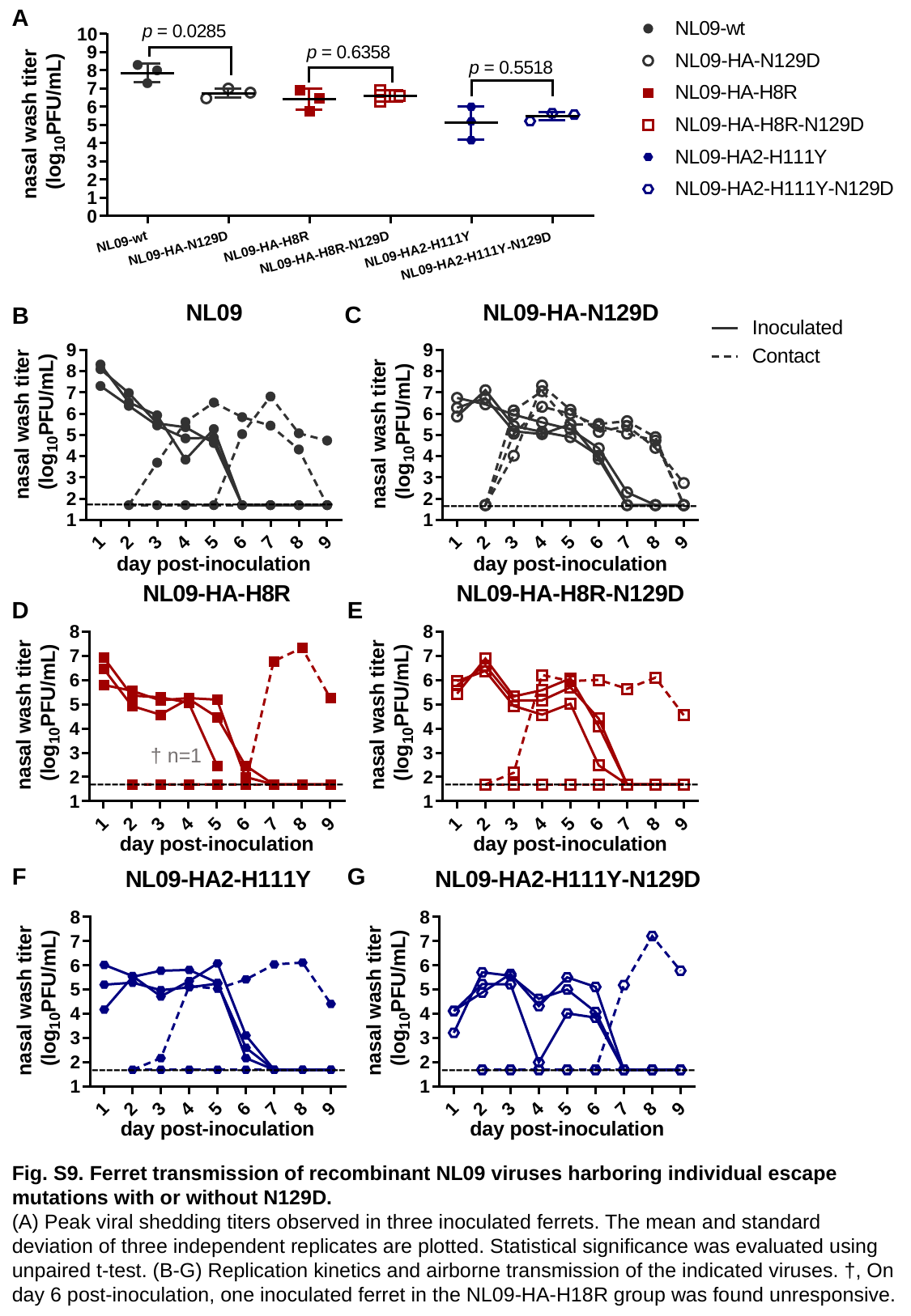

A
C
B
D
E
† n=1
F
G
Fig. S9. Ferret transmission of recombinant NL09 viruses harboring individual escape mutations with or without N129D.
(A) Peak viral shedding titers observed in three inoculated ferrets. The mean and standard deviation of three independent replicates are plotted. Statistical significance was evaluated using unpaired t-test. (B-G) Replication kinetics and airborne transmission of the indicated viruses. †, On day 6 post-inoculation, one inoculated ferret in the NL09-HA-H18R group was found unresponsive.
